# Pattern learning reveals brain asymmetry to be linked to socioeconomic status

**DOI:** 10.1093/texcom/tgac020

**Published:** 2022-05-20

**Authors:** Timm B Poeppl, Emile Dimas, Katrin Sakreida, Julius M Kernbach, Ross D Markello, Oliver Schöffski, Alain Dagher, Philipp Koellinger, Gideon Nave, Martha J Farah, Bratislav Mišić, Danilo Bzdok

**Affiliations:** Department of Psychiatry, Psychotherapy and Psychosomatics, Faculty of Medicine, RWTH Aachen University, Aachen, Germany; Department of Health Management, School of Business, Economics and Society, Friedrich-Alexander-Universität Erlangen-Nürnberg, Nürnberg, Germany; Department of Biomedical Engineering, McConnell Brain Imaging Center (BIC), Montreal Neurological Institute (MNI), Faculty of Medicine, School of Computer Science, McGill University, Montreal, Quebec, Canada; Department of Psychiatry, Psychotherapy and Psychosomatics, Faculty of Medicine, RWTH Aachen University, Aachen, Germany; Department of Neurosurgery, Faculty of Medicine, RWTH Aachen University, Aachen, Germany; McConnell Brain Imaging Center (BIC), Montreal Neurological Institute (MNI), Faculty of Medicine, McGill University, Montreal, Quebec, Canada; Department of Health Management, School of Business, Economics and Society, Friedrich-Alexander-Universität Erlangen-Nürnberg, Nürnberg, Germany; Montreal Neurological Institute (MNI), McGill University, Montreal, Quebec, Canada; Department of Economics, School of Business and Economics, Vrije Universiteit Amsterdam, Amsterdam, The Netherlands; La Follette School of Public Affairs, University of Wisconsin-Madison, Madison, WI, USA; Marketing Department, The Wharton School, University of Pennsylvania, Philadelphia, PA, USA; Center for Neuroscience and Society, University of Pennsylvania, Philadelphia, PA, USA; McConnell Brain Imaging Center (BIC), Montreal Neurological Institute (MNI), Faculty of Medicine, McGill University, Montreal, Quebec, Canada; Department of Biomedical Engineering, McConnell Brain Imaging Center (BIC), Montreal Neurological Institute (MNI), Faculty of Medicine, School of Computer Science, McGill University, Montreal, Quebec, Canada; Mila – Quebec Artificial Intelligence Institute, Montreal, Quebec, Canada

**Keywords:** brain lateralization, hemispheric asymmetry, machine learning, multi-output pattern learning, population neuroscience, socioeconomic status

## Abstract

Socioeconomic status (SES) anchors individuals in their social network layers. Our embedding in the societal fabric resonates with habitus, world view, opportunity, and health disparity. It remains obscure how distinct facets of SES are reflected in the architecture of the central nervous system. Here, we capitalized on multivariate multi-output learning algorithms to explore possible imprints of SES in gray and white matter structure in the wider population (*n* ≈ 10,000 UK Biobank participants). Individuals with higher SES, compared with those with lower SES, showed a pattern of increased region volumes in the left brain and decreased region volumes in the right brain. The analogous lateralization pattern emerged for the fiber structure of anatomical white matter tracts. Our multimodal findings suggest hemispheric asymmetry as an SES-related brain signature, which was consistent across six different indicators of SES: degree, education, income, job, neighborhood and vehicle count. Hence, hemispheric specialization may have evolved in human primates in a way that reveals crucial links to SES.

## Introduction

The notion of socioeconomic status (SES) describes an individual’s embedding in the social hierarchy. SES is defined as a measure of combined economic and social status (Baker 2014; House 2002; Galobardes et al. 2006). In sociology, SES is regarded a latent construct and is often measured using a composite measure of education, income, and occupation or a variation of these indicators (Baker 2014). SES shapes our approach to everyday social interaction by gating access to various resources including friendship networks, nutrition, as well as education and culture (Adler et al. 1994; Krieger et al. 1997). In particular, SES interferes with ready access to health services and is linked to general health satisfaction. Low SES escalates the risk of mental disease, suicide, and various physical diseases such as diabetes and obesity (Sareen et al. 2011; Stringhini et al. 2017). Given socioeconomic differences in the distribution of health-relevant behaviors such as smoking and physical inactivity (Stringhini et al. 2017), some diseases are thought to be a consequence of scarce socioeconomic capital. Nonetheless, the biological basis that underlies SES-related behavioral dispositions remains poorly understood (Pampel et al. 2010).

More recent work has transitioned from investigating factors that are environmental or epidemiological in nature to interindividual differences in biologic determinants of SES. The advent of concerted biobank initiatives has now opened the door to study the associations between idiosyncratic biological features and SES traits that condition everyday life (e.g. Tyrrell et al. 2016). For instance, a genome-wide association study (GWAS) in 112,151 participants from the UK Biobank (UKBB) reported that common-variant genetic profiles account for 11% of household income and 21% of interindividual differences in social deprivation (Hill et al. 2016). The authors thus concluded that some SES-related genetic variants are associated with genes that are preferentially expressed in the central nervous system (Hill et al. 2016).

The present population-scale brain-imaging investigation takes the next natural step in beginning to complete principles of how SES is manifested in the human brain. The thrust of this endeavor is grounded in the carefully documented relationship between SES and various dimensions of cognitive abilities (Hackman and Farah 2009). Intellectual capacities are an important predictor of and share genetic underpinnings with different health outcomes (Henderson et al. 2012; Hagenaars et al. 2016; Marioni et al. 2018). We are committed to making concrete steps toward filling this knowledge gap by unraveling key neural substrates that explain interindividual differences in SES. Such research agenda has recently been advocated in the neuroscience community (Farah 2018). Additionally, the explanatory grip of many existing studies on the brain-SES correspondence has been limited by relatively small participant samples with less than ≈1000 subjects. It remains to be seen which previous findings from in-laboratory studies faithfully generalize to real-world settings and to the broader human population.

Here, we respond to recent calls for a population-scale approach to SES (Farah 2018) by data-mining the brain-imaging resources from several thousand UKBB participants. To go beyond previous neuroscience studies on isolated SES factors, we adopt a different-in-kind approach by bringing multivariate multi-output models from machine learning to population brain-imaging of gray and white matter anatomy. These algorithmic tools optimally exploit relationships between 6 key SES measures to obtain more robust model fits, which have yielded stronger generalization performance to new participants (Rahim et al. 2017; Bzdok and Meyer-Lindenberg 2018). Structural brain measures are found to be relatively more state-independent, compared with functional brain measures, which is important for our goal of capturing relatively more time-enduring behavioral tendencies. SES indeed corresponds to a set of characteristics that tend to be relatively stable across time, place, and context. Yet, shifts in SES or social mobility were reported to be associated with changes in different brain features (Weissman et al. 2018; Dufford et al. 2020).

## Material and methods

### Human population data resource

The UKBB is a prospective epidemiological resource that provides rich information including brain imaging, genetic, and various biological and lifestyle measurements in a cohort of ∼500,000 participants recruited from across Great Britain (https://www.ukbiobank.ac.uk/). Among the brain imaging data of the 9935 participant UKBB release (see Supplementary Table 1 regarding demographic information), we focused on high-resolution *T*_1_-weighted structural brain scans as a measure of whole-brain gray matter morphology as well as diffusion-weighted brain scans reflecting white matter microstructure (Miller et al. 2016). Participants were recruited at ages 40–70, with the brain-imaging supplemented administered at 28–30 months after baseline assessment on average. For the sake of reproducibility and comparability, all our tests of brain–behavior association were based on the precomputed and vetted image-derived phenotypes (Miller et al. 2016). For our analyses of gray matter structure, we relied on volume estimates in 111 cortical and subcortical regions defined by the Harvard-Oxford atlas as part of UKBB Imaging. For our analyses of white matter structure, we relied on estimates of fiber bundle fractional anisotropy in 48 tracts defined by the Johns Hopkins atlas. All structural magnetic resonance imaging (MRI) data were preprocessed using the pipelines and quality-control workflows by the FMRIB team, Oxford University, UK (Alfaro-Almagro et al. 2018). The uniform preprocessing increases the comparability of our findings to other and future UKBB studies. In a preparatory step, we used common linear deconfounding to remove variation in all brain-imaging-derived phenotypes that could be explained by interindividual differences in head size or body mass index, following previously published UKBB research (Kernbach et al. 2018). As such, effects emerging from the subsequent modeling steps on the thus cleaned brain phenotypes cannot be explained by differences in brain size or adiposity. All participants provided informed consent. Further information on the consent procedure can be found elsewhere (http://biobank.ctsu.ox.ac.uk/crystal/field.cgi?id=200).

### Target measures of SES from the UK biobank

Building on previous (non-brain-imaging) research on SES in the UKBB cohort (Tyrrell et al. 2016), our study used 6 different indices that capture aspects of SES. If necessary, the underlying scale was inverted for our analyses. As such, a higher value stands for a higher level of SES in all measures (cf. Table 1).

1) *Education Years* (data-field 845): This variable indirectly measures the years of training of the participants. The age that they completed continuous full-time education is provided in years. The variable provides the age when school education was finished, which does not include experience during college or university.
2) *Degree* (data-field 6138): This variable specifies the highest educational achievement, ranging from none to professional qualifications to college or university qualification.
3) *Income* (data-field 738): This variable indexes the average total household income before tax in £, ranging from <£18,000 to >£100,000 annual income.
4) *Job* (data-field 132): This variable captures the participants’ current employment according to the Standard Occupational Classification 2000 (Statistics USBoL 1999). We have encoded this variable as knowledge worker (“white collar”) vs. manual worker (“blue collar”) traits.
5) *Vehicle count* (data-field 728): This variable specifies the number of vehicles that are available in a household as a measure of material wealth.
6) *Neighborhood level* (data-field 189): This variable is defined based on the Townsend Deprivation Index (Townsend et al. 1988). This established metric captures the aspects of deprivation such as reflected in unemployment, household overcrowding, non-car ownership, and non-home ownership. We inverted this demographic index such that a higher number reflects a higher SES.

**Table 1:** Behavioral links of age and sex to the six SES dimensions. Univariate regression of a given SES dimension onto age or sex of UK biobank participants (cf. methods).

	Age	Sex
Income	−0.26	0.07
Education_years	−0.05	0.02
Degree	−0.10	0.02
Neighborhood_level	-0.10	0.01
Vehicle_count	−0.07	0.02
Job	−0.22	0.11

To quantify the extent to which these SES dimensions carry complementary dimensions that underlie socioeconomic diversity of the population, we computed several characterizations of their interrelationship. First, we conducted a cross-correlation analysis of the 6 SES dimensions that quantified the degree of linear association between all possible pairs of SES indicators. The linear association strength was measured by computing Pearson’s correlation coefficient. Second, we carried out a mixture decomposition of the 6 SES dimensions by means of principal component analysis. This complementary analysis investigated the extent that any possible combinations of SES indices may conjointly carry similar information about participants’ behavior.

As variables of potential confounding influence for our analyses, we introduced terms that capture participant age (data-field 21,022), sex (data-field 31), non-linear age-sex interactions (age^2^, age^*^sex, and age^2^^*^sex), and fluid intelligence summary score (data-field 20,016). Introducing handedness (data-field 1707) as an additional variable of no interest led to virtually identical results and hence the same neuroscientific conclusions.

### Workflow for multi-output pattern analysis via transfer learning

To analyze population-level brain variation with regards to 6 SES dimensions in an integrated model estimation, we capitalized on multi-output ridge regression (Caruana 1998; Simila and Tikka 2007; Borchani et al. 2012; Hastie et al. 2015; Rahim et al. 2017; Bzdok and Meyer-Lindenberg 2018). Based on our careful benchmarking of several popular machine learning tools (cf. Supplementary Material and Methods), we have committed to the linear modeling solution, given that this was the overall most predictively successful model class, for all subsequent analyses on the brain-SES correspondence (Tables 2 and 3). Fitting a supervised pattern-learning algorithm to jointly explain variation in several output variables yields modeling advantages, if these target phenotypes share a degree of mutual relatedness. Importantly, single model solution results from relating brain phenotypes of brain gray matter or white matter measures to all 6 SES dimensions in a principled fashion. This modeling strategy is distinct from the ordinary approach of fitting a separate linear regression model to each SES dimension in an independent modeling step, which would ignore the 5 remaining SES dimensions in each instance. Additionally, sharing statistical strength between related output dimensions is known to yield more robust modeling solutions. Profiting from such “sharing of statistical strength” can lead to more precise point estimates for the modal parameter values. As a third asset, a ridge-regression-based approach also suggested itself because of the native ability of this quantitative tool to effectively handle possible auto-correlation among the input variables. Indeed, the technical literature has stated that “If N>D [more subjects than input variables], but the variables are correlated, it has been empirically observed that the prediction performance of ridge is better than that of lasso” (Murphy 2012).

**Table 2:** Benchmarking analysis of different machine-learning algorithms regarding out-of-sample prediction from gray matter volumes.

	Pearson’s *r*	Explained Variance Score	Mean Absolute Error	Coefficient of Determination	Mean Squared Error
Ridge Regression	0.2334	0.0599	0.7967	0.0591	0.9421
Random Forest	0.2323	0.0613	0.8016	0.0601	0.9410
Gradient Boosting	0.2310	0.0603	0.8013	0.0591	0.9420
k-Nearest Neighbor	0.1223	0.0019	0.8233	-0.0012	1.0021
Kernel Ridge Regression	0.2256	0.0561	0.8011	0.0550	0.9461
Deep Learning	0.1911	0.0374	0.8080	0.0363	0.9648

(cf. Supplementary Table 11)

**Table 3:** Benchmarking analysis of different machine-learning algorithms regarding out-of-sample prediction from white matter microstructure.

	Pearson’s *r*	Explained Variance Score	Mean Absolute Error	Coefficient of Determination	Mean Squared Error
Ridge Regression	0.2261	0.0562	0.7954	0.0551	0.9394
Random Forest	0.2186	0.0552	0.7949	0.0539	0.9319
Gradient Boosting	0.2202	0.0559	0.7944	0.0546	0.9312
k-Nearest Neighbor	0.1395	0.0099	0.8140	0.0077	0.9775
Kernel Ridge Regression	0.2222	0.0553	0.7927	0.0541	0.9318
Deep Learning	0.1980	0.0406	0.7969	0.0393	0.9464

(cf. Supplementary Table 12)

In a transfer learning approach, multi-task ridge regression solved the following numerical optimization objective for the ~10,000 participant sample (Hastie et al. 2015):$\hat{W}={argmin}_{(W\in{\mathbb{R}}^{p\ x\ k})}\ \frac{1}{2\ast n}\ {\Big\Vert Y- XW\Big\Vert}_F^2+\frac{1}{2}\ast \alpha \ast{\Big\Vert W\Big\Vert}_F^2$, where $Y\in{\mathbb{R}}^{nxk}$ denotes the vector-valued response with $k$ outcome components (z-scored across participants) that captured the 6 SES indicators from the $n$ participants, $X\in{\mathbb{R}}^{n\times p}$ denotes the matrix holding the collection of input variables for all participants $n$ with a number of input variables $p$, and $W\in{\mathbb{R}}^{p\times k}$ is the matrix of $p\ast k$ slope parameters to be estimated, $\alpha$ is the regularization strength of the ${\ell}_2$-penalty term (defaulted to α = 0.01), while ${\Big\Vert A\Big\Vert}_F=\sqrt{\sum_{i=1}^q{\sum}_{j=1}^r{\Big|{a}_{ij}\Big|}^2}$ denotes the Frobenius matrix norm. This modeling framework enabled adaptively borrowing information between the model parameters to exploit that the 6 SES estimation problems are different but mutually related. One outcome estimation induced movement in the estimation of the respective $k-1$ other outcome estimations. Thus, learning 1 brain-SES pattern helped in learning the other brain-SES associations. This instance of parameter sharing imposed group structure in the $k$ coupled model estimation goals (i.e. called “tasks” in the machine learning community). In this way, regressing interindividual variation in 1 SES dimension against the set of brain features affected how the other SES dimensions were regressed onto these brain-imaging-derived measurements. The pooled regularized loss for multi-output learning was minimized using the coordinate descent solver.

The input brain phenotypes were either 111 region volume measures from the Harvard-Oxford atlas or 48 fiber tract microstructural measures from the Johns Hopkins atlas (Miller et al. 2016; Alfaro-Almagro et al. 2018) (cf. above). This analysis scenario implies joint estimation of $p$ = 111 + number of control variables or $p$ = 48 + number of control variables, respectively. As preliminary de-confounding procedure, any (linear) variation in these brain phenotypic measures that could be explained by variation in head size or body mass index was removed from the brain variables before submitting these variables to the model of interest, following previous UKBB research (Kernbach et al. 2018; Kiesow, Spreng, et al. 2020). Before estimation of the pattern-extraction model, each input and output variable was z-scored across participants by de-meaning to zero average and unit-variance scaling to one.

To improve the statistical quality of the parameter weights learned by the pattern-learning algorithm, we derived population confidence intervals around each model parameter value using an instance of bootstrapping aggregation (Efron and Tibshirani 1994). In each of 100 bootstrapping iterations, we have resampled among the ≈10,000 UKBB participants a bootstrap dataset with the same number of participants. Based on each of these drawn bootstrap datasets, the identical multi-output algorithm fitting procedure was repeated. This analysis yielded 100 candidates for each parameter value that we have averaged across participants to obtain a more solid final multi-output modeling solution.

### Nonparametric permutation procedure to test for significant regional effects

We determined the statistical significance of each region-SES and each tract-SES population association by means of nonparametric perturbation testing. The impact of a given set of brain phenotypes, either gray matter volumes or white matter tract microstructure, on associations with SES dimensions was isolated by shuffling the participants set of 6 SES measures. This randomization issued a perturbed set of unrelated brain phenotypes and SES measures to selectively destroy the correspondence of brain region or tract variation with the SES dimension across participants.

A compilation of 1,000 such perturbed datasets was thus generated from the original participant sample. In each of the 1,000 perturbed versions of the original dataset, the identical multi-output ridge regression pipeline was carried out and evaluated in the same fashion. Relying on minimal modeling assumptions, a valid empirical null distribution was derived for the brain phenotype parameters obtained from multi-output regression analysis. In 1,000 permutation iterations, the matrix of brain phenotypes was held constant, while the sets of SES measures were subject to participant-wise random shuffling. The permuted surrogate data preserved the statistical structure idiosyncratic to the volumetric or diffusion MRI signals, respectively. Yet, this empirical null model permitted to selectively eliminate the signal property related to the brain’s association with SES measures to be tested (Efron 2012). The thus generated distribution reflected the null hypothesis of absent association between brain region/tract variation and SES traits across individuals. *P*-values were obtained based on counting the number of times that the original (bagged, cf. above) model parameter values were smaller or bigger than those estimated from the null multi-output model, where multiple comparisons were explicitly taken into account by searching through a space of multi-output algorithm solutions [all *P* < 0.001, family-wise error corrected (Kernbach et al. 2018; Karrer et al. 2019; Spreng et al. 2020)].

### Functional profiling of the structural brain patterns underlying SES

To obtain a data-driven functional characterization of the spatial pattern of derived brain-SES associations, we capitalized on the Neurosynth resource. This is one of the largest existing repositories of functional brain-imaging results with rich experimental descriptions (see Supplementary Table 14 for list of 123 terms). We obtained probabilistic measures of the association between our gray matter atlas (cortical and subcortical regions, cf. above) and terms of neurocognitive processes from Neurosynth. This meta-analytic tool synthesized results from >15,000 published functional brain-imaging studies by searching for high-frequency key words (such as “pain” and “attention”) that are published alongside standardized coordinates of neural activity responses [https://github.com/neurosynth/neurosynth (Yarkoni et al. 2011)]. Neurosynth measured associations as the probability that a given term is reported in a brain-imaging experiment if neural activity changes are observed at a given brain location. The approach is based on the co-occurrence that certain brain areas are frequently mentioned in conjunction with certain words. We focused primarily on cognitive function and therefore limited the terms of interest to cognitive and behavioral terms rather than clinical or diagnostic concepts. These terms were aligned with the Cognitive Atlas, a public ontology of cognitive science (Poldrack et al. 2011), resulting in 123 terms ranging from umbrella terms (e.g. “attention,” “emotion”) to specific terms of cognitive processes (e.g. “visual attention,” “episodic memory”), behaviors (e.g. “eating,” “sleep”), and emotional states (e.g. “fear,” “anxiety”) (Supplementary Table 14). The location-by-term associations reported by Neurosynth were parcellated into 111 cortical and subcortical regions (see above), yielding a region-by-term matrix that quantitatively represents how regional functional activity is related to mental processes.

Parcellated data from Neurosynth were correlated with brain-wide parameter estimates for each SES variable. That is, each column of the location-by-term matrix obtained from Neurosynth was independently correlated with the regional parameter values generated from our multi-output SES model. Correlations highlight the spatial overlap between functional activations associated with a given cognitive term and the obtained whole-brain correlates of SES.

### Scientific computing implementation

Python was selected as the scientific computing engine. Capitalizing on its open-source ecosystem helps enhance replicability, reusability, and provenance tracking. The *scikit-learn* package provided efficient, unit-tested implementations of state-of-the-art machine learning algorithms (http://scikit-learn.org/). This general-purpose machine-learning library was interfaced with the *nilearn* library for design and efficient execution of brain-imaging data analysis workflows (http://github.com/nilearn/nilearn).

## Results

### Several indices of SES tell rich and complementary stories

To assess complementary perspectives on how UK Biobank participants are positioned in the social hierarchy, we have mapped a portfolio of six different SES measures to the brain (Oakes and Rossi 2003). Our UKBB sample comprised 48% men and 52% women who were aged 40–70 years at recruitment, with British (92%), Irish (3%), other white (2%), or other ethnic (3%) background. More details on the population characteristics are openly accessible to anybody elsewhere (https://biobank.ndph.ox.ac.uk/showcase/). Following previous studies on the UKBB population cohort (Tyrrell et al. 2016), we have built on the participant profiling including 6 indicators of SES (see methods section for further details).

**Fig. 1:**
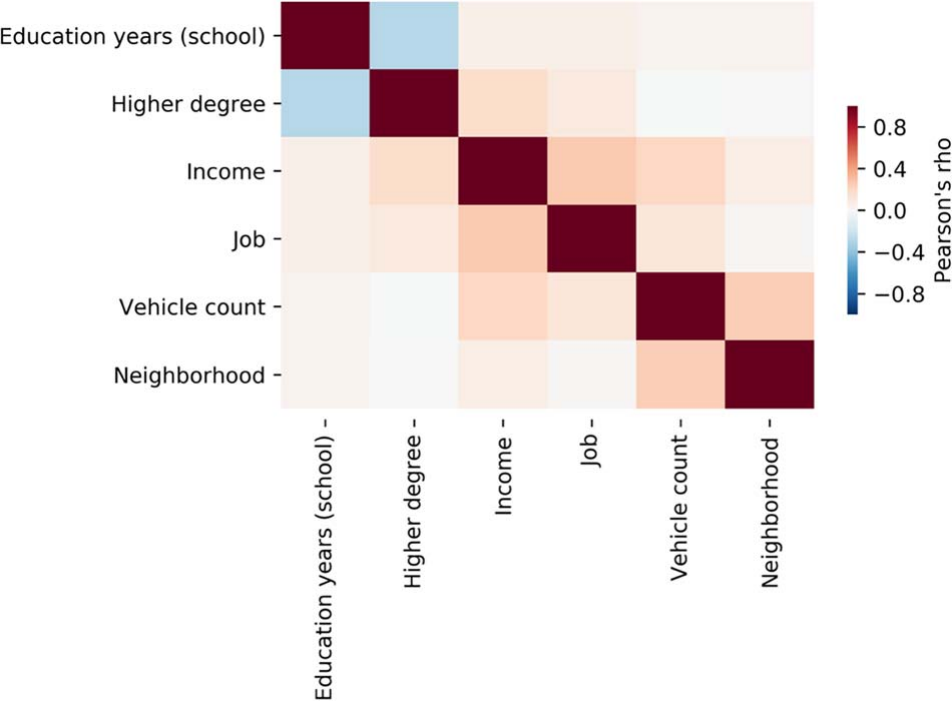
Mutual relationships between different indicators of SES. This exploratory analysis in 10.000 UK biobank participants shows that socioeconomic traits relate to a complex constellation of social, demographic, and financial aspects. Pearson’s correlation shows that these factors are moderately interrelated. The behavioral markers thus capture largely complementary aspects of an individual’s standing in society (Farah 2018). A negative relation between the indices of higher degree and education years is expected since education years only count time in school (not college or university) (see Materials and Methods section). The color tones indicate Pearson’s correlation coefficients ϱ. All index–index relations yielded a ϱ < 0.5. These descriptions thus indicate that our SES dimensions involve mostly specific, but also a degree of shared variation across participants. For mixture deconvolution of joint and distinct information in the SES indicators, see Supplementary Fig. 1.

In initial exploratory steps, we conducted descriptive summaries of these demographic and lifestyle indicators. These preparatory analyses quantified the degree to which the examined sources of population variation carry partly separate information that underlies an individual’s SES profile (Fig. 1). At the behavioral level, all pairwise Pearson correlation coefficients were computed for the 6 target dimensions of SES. We thus assessed the strength of linear association between each possible combination of 2 SES indicators. All pairwise index-index relations yielded a certain extent of linear relation, but all with a Pearson’s ϱ < 0.5. In other words, the 6 target SES measures were far away from being identical. Yet, these trait markers carry a certain degree of shared information as evidenced by the estimated Pearson correlation coefficients.

Next, we interrogated how combinations of multiple SES indicators may show driving effects in decomposing the SES construct as measured in UKBB participants. We computed a mixture deconvolution of the 6 examined SES dimensions by means of principal component analysis (Supplementary Fig. 1). The overall variance across the SES indicators was largely explained by distinct latent principal dimensions of variation that mostly involved dominant contributions from single SES indicators. The most prominent joint variation was identified by this matrix decomposition technique between *income* and several other SES indicators (32.2% explained variance). The second most explanatory pattern made apparent joint variation between the number of education years and the achieved highest degree (22.2% explained variance). The third pattern highlighted especially opposite variation for neighborhood and job type (15.4% explained variance). Each of the first 5 patterns discovered from the set of SES indicators explained at least 10% of the overall variation (for full details see Supplementary Fig. 1). As such, this second descriptive analysis confirmed that no 2 indicators carried identical or largely redundant information about the SES of the UKBB participants.

Taken together, this preliminary exploration of our target SES dimensions in the participants of the 10,000 UKBB release invigorated that the 6 measures tell rich and partly separate stories about how the participants differ in their socioeconomic standing in society. This behavioral finding suggests specific brain correlates and a coherent joint pattern of SES dimensions that are robust at the population level.

### Differences in SES indices are linked to gray matter morphology

We sought to simultaneously probe association with 6 SES dimensions in an integrated modeling framework (Rahim et al. 2017; Bzdok and Meyer-Lindenberg 2018). For this purpose, we brought to bear a supervised algorithm to extract principled patterns from variation in gray matter volume from 111 target regions (Harvard-Oxford atlas). This multi-output learning algorithm (i.e. L2-penalized multi-task learning, see methods section for details) allowed us to tease apart coherent multivariate patterns of how the set of brain features are related to the 6 SES dimensions in a single modeling process (see methods section).

**Fig. 2:**
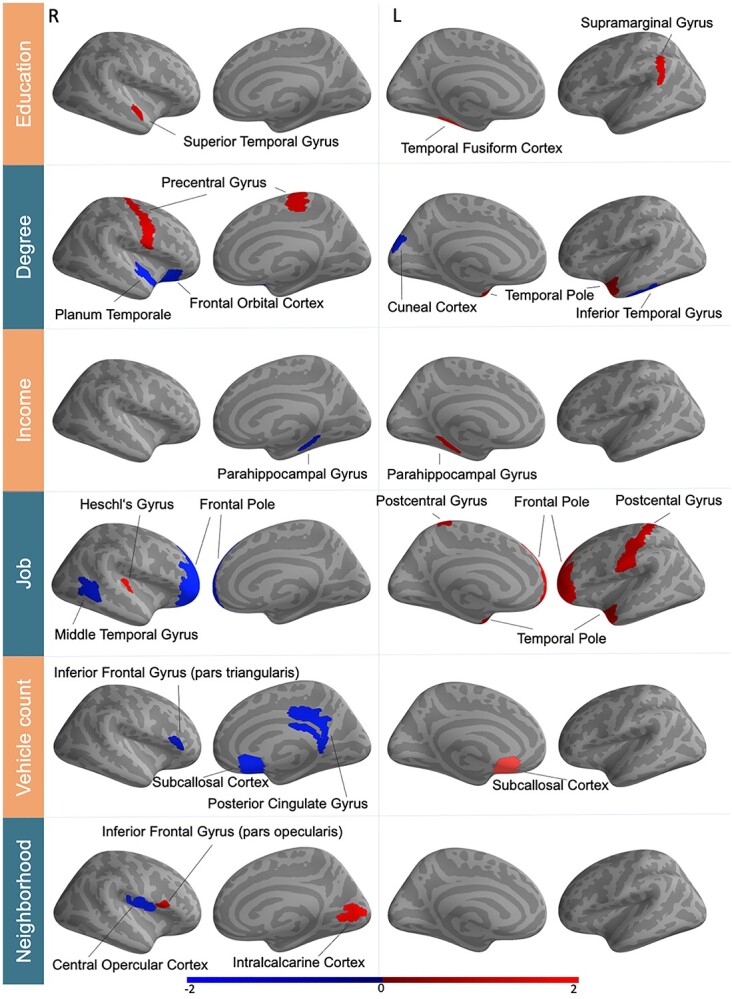
Population variability in SES shows imprints in regional gray matter morphology. Supervised learning algorithms identified a variety of multivariate patterns between 111 gray matter regions and SES indicators. These brain–behavior associations (all significant at *P* < 0.001, after explicitly considering multiple comparisons) uncovered both positive (red) and negative (blue) direction. Certain regions, including the left caudate and left temporal pole in particular, were associated with several SES determinants. These brain manifestations uncover similarity and idiosyncrasies between the six examined SES indicators. The color bar represents z-scores. R/L = right/left hemisphere. For full effect sizes and bootstrap uncertainty intervals used to assess significance, see Supplementary Tables 2–4.

This analytical approach showed that the SES dimensions were significantly (*P* < 0.001) associated with gray matter variation in both cortical and subcortical regions (Fig. 2; Supplementary Figs 2 and 3, Supplementary Tables 2–4). The cortical regions with relevant association strengths tapped on all four major lobes of the cerebral cortex, as well as both the left and right hemisphere. The subcortical regions with robust links to interindividual differences in SES dimensions included the caudate nucleus and brainstem.

Several gray matter regions were consistently associated with more than 1 of the 6 SES determinants. For instance, a brain-SES association was evident for the volume of the left caudate nucleus that was positively related to *degree* (0.077 ± 0.048/0.109 [5%/95% confidence interval formed based on 100 participant resampling iterations by means of the bootstrap]) and *education* (0.075 ± 0.035/0.119). As another example, the left temporal pole showed a positive SES association with both *degree* (0.058 ± 0.035/0.084) and *job* (0.046 ± 0.016/0.075).

### Differences in SES indices are linked to white matter microstructure

**Fig. 3:**
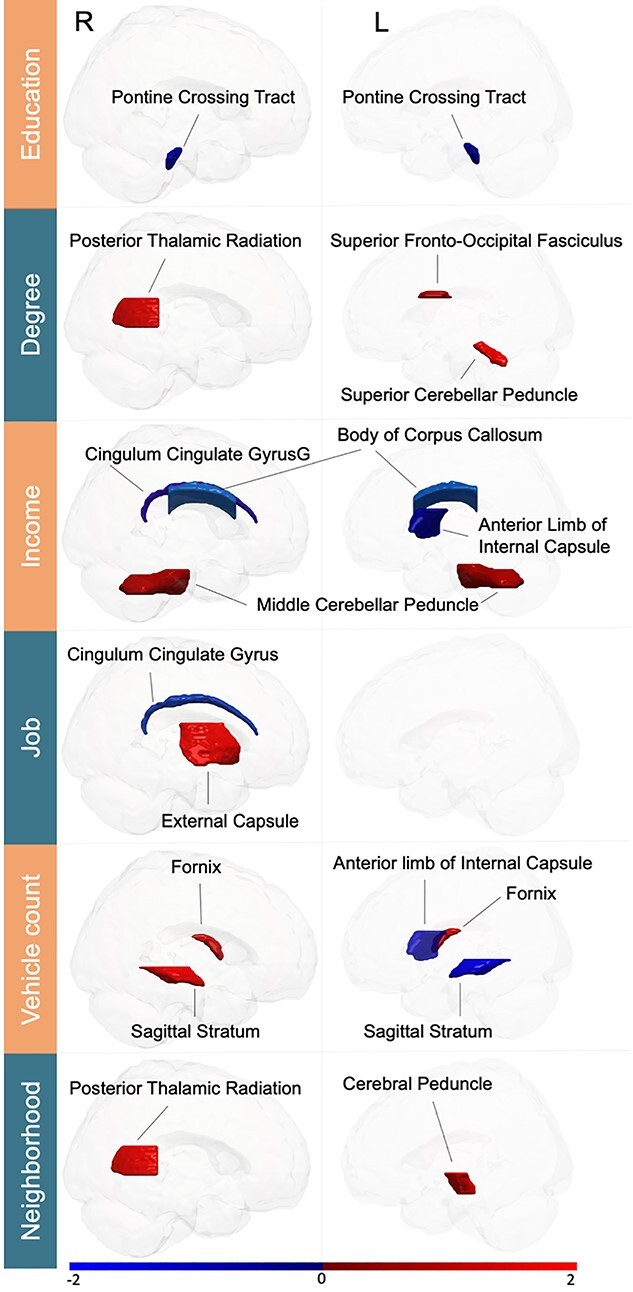
Population variability in SES reverberates in white matter tract microstructure. Supervised learning algorithms revealed associations in positive (red) and negative (blue) direction specific to SES indicators across 48 white matter tracts. The identified links to SES involved association, commissural, and projection fibers (all statistically significant at *P* < 0.001, after explicitly considering multiple comparisons). Three fiber tracts (right cingulum and posterior thalamic radiation, left internal capsule) were robustly associated with multiple SES dimensions. Analogous to our gray matter findings (Fig. 2), these brain substrates of SES in white matter corroborate a complementary composition of single SES dimensions. The color bar represents z-scores. R/L = right/left hemisphere. For full effect sizes and bootstrap uncertainty intervals used to assess significance, see Supplementary Tables 5–7.

In analogous fashion, we subsequently deployed the supervised multi-output learning algorithm on population variation in microstructural properties (mean fractional anisotropy) in 48 target white matter tracts (Johns Hopkins University atlas). The investigated SES dimensions showed a rich pattern of association with interindividual variation of major white matter tracts. The brain-SES associations that achieved statistical significance (*P* < 0.001) included anatomical connections of the brain, such as tracts between cerebrum, diencephalon, and cerebellum (Fig. 3, Supplementary Fig. 3, Supplementary Tables 5–7). Similar to our findings in gray matter (cf. above), the brain-SES associations revealed a differentiated spatial pattern spread across both brain hemispheres. Moreover, SES dimensions revealed significant effects in several kinds of anatomical white matter tracts, which included association, commissural, and projection fibers (Supplementary Table 7).

In particular, several fiber tracts were associated with more than one SES factor, similar to our region associations with SES (cf. above). For instance, fractional anisotropy of the right cingulum was negatively linked to *incom*e (−0.057 ± −0.081/−0.032 [5%/95% confidence interval formed based on 100 participant resampling iterations by means of the bootstrap]) and *job* (−0.069 ± −0.097/−0.038). As another example, brain-SES associations became apparent between fractional anisotropy of the anterior limb of the left internal capsule for *income* (−0.081 ± −0.110/−0.053) and *vehicle count* (−0.066 ± −0.100/−0.034). Moreover, the right posterior thalamic radiation also demonstrated 2 positive brain-SES associations for *degree* (0.088 ± 0.056/0.121) and *neighborhood level* (0.072 ± 0.038/0.103).

Taken together, a brain-wide pattern of gray matter regions and white matter tracts was reliably linked to more than 1 of the examined SES dimensions. Notably, there was no single region or major fiber tract that we observed to be associated with one SES variable in one direction and with another SES variable in the opposite direction. In other words, all our findings in architectural features of gray and white matter were directionally consistent. These brain-imaging findings complement our exploratory analyses of the distinctness and commonality within SES dimensions in our UKBB participant sample (see above). In line with this, the distributed pattern of these associations highlights distinct, but partly overlapping brain signatures of SES dimensions.

### Hemispheric asymmetry characterizes the brain representation of SES

The distributed pattern of brain-SES associations was suggestive of a global motif of lateralization effects. Among all gray matter regions in our atlas, the caudate nucleus, frontal pole, posterior parahippocampus, as well as the subcallosal cortex showed associations with SES in both brain hemispheres. Yet, all of these associations turned out to be positive in the left brain but negative in the right brain (cf. Supplementary Tables 2–4). As such, positive SES associations in brain features appeared to dominate in the left hemisphere. In contrast, negative SES associations appeared to dominate in the right hemisphere.

Therefore, we directly examined the possibility of a coherent brain-wide lateralization pattern in SES using dedicated statistical tests (Supplementary Fig. 3). Indeed, we confirmed systematically more positive than negative SES association counts with cortical gray matter regions in the left hemisphere and a diametrically opposed pattern of brain-SES associations for the right hemisphere (χ^2^ = 4.991; df = 1; *P* = 0.025). This hemispheric lateralization effect did not reach statistical significance in anatomical fiber tracts (χ^2^ = 0.202; df = 1; *P* = 0.653) in this approach.

**Fig. 4:**
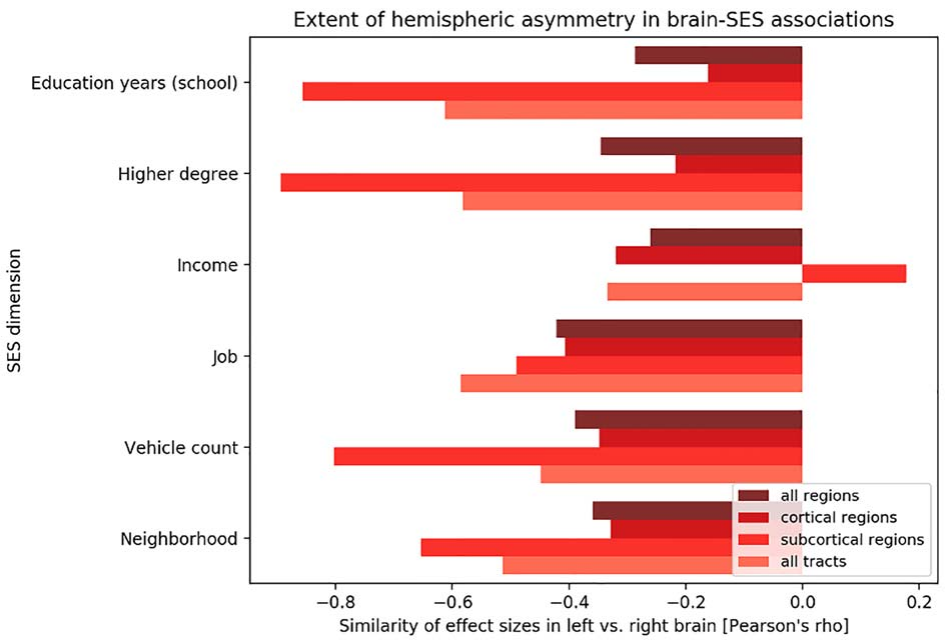
The left and right brain hemisphere relate to SES factors in opposite ways. All main analyses in our study were based on multivariate multi-output algorithms of six major determinants of SES on brain atlas features. Separate pattern-learning analyses were conducted to explain the SES dimensions based on either a) gray matter volumes of 111 cortical and subcortical regions (Harvard-Oxford atlas; Fig. 2) or b) microstructure of 48 white matter tracts (Johns Hopkins University atlas; Fig. 3). After model building, the formed estimates for the model parameters were summarized for interrogation of systematic interhemispheric effects. To this end, we computed Pearson’s correlation coefficients across the (bootstrap-uncertainty-adjusted) model parameters of brain features that are homologous in the left vs. right brain (*x*-axis). This post-hoc aggregation across the computed models uncovered notable anti-correlation in the associations of how left-sided and right-sided brain features are linked to interindividual variation in SES. Our observation of hemispherically differentiated brain-SES correspondence held up when considering a) all regions (darkest red tone), b) only cortical regions, c) only subcortical regions, and d) only fiber tracts (lightest red tone). When restricting attention to statistically significant brain-SES associations, rather than the spatial distribution of the full effect sizes, we substantiated evidence for a distributed brain asymmetry pattern of SES (Supplementary Fig. 4).

In a second post-hoc analysis of the obtained multi-output modeling solution, we examined more closely the obtained SES-related effect sizes and parameter uncertainty in the left vs. right brain. To this end, we quantified the extent of hemispheric asymmetry in how the estimates of model parameter values corresponding to specific gray matter regions and white matter fiber tracts were associated with the 6 SES measures (Supplementary Tables 2 and 5). Based on continuous effect sizes, a consistent trend became apparent for opposite regression weights in the left vs. right hemisphere (Fig. 4). This observation was reliable whether we included IQ as covariate in our multi-output learning model or not. Moreover, the observation was largely consistent across subanalyses of only cortical regions, only subcortical regions, only fiber tracts, as well as across the 6 examined SES dimensions (Fig. 4).

Across 2 complementary post-hoc inspections of the obtained brain-SES associations, we ascertained a global pattern of hemispheric asymmetry based on measures of gray matter morphology and fiber tract microstructure in the UKBB cohort. We add support for a pattern of left–right asymmetry in brain-SES associations when resorting to classical linear regression, without multi-output modeling and without l2-penalization shrinkage (Supplementary Tables 8 and 9). This hemispheric asymmetry pattern was observed again when applying classical linear regression to the principal components derived from our 6 SES indicators (Supplementary Tables 10 and 11). Moreover, we have replicated the left–right asymmetry in 3 independent samples of 10,000 new UK Biobank participants in an external validation check (Supplementary Figs 4–8).

### Functional profiling of the brain manifestation of SES indices

**Fig. 5:**
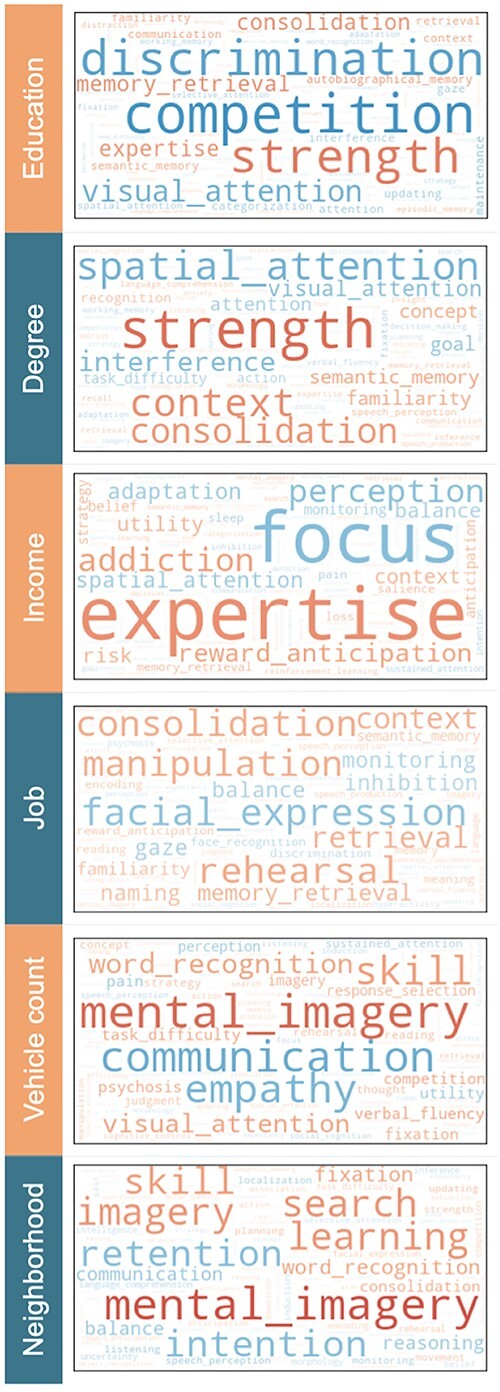
Functional annotation of how SES is linked to the measures of brain architecture. A large-scale database of brain-imaging experiments—Neurosynth—was queried for co-occurrence with ontological terms that map onto the derived gray-matter-wide patterns of SES associations. We computed the similarity between a given term’s functional activity patterns and the obtained brain correlates of SES (*red/blue* = positive/negative correlation, range = [−0.3, 0.3]). Word size represents the relative magnitude of each brain-concept association. We have also performed this meta-analytic profiling analysis separately for each hemisphere (Supplementary Fig. 9) and for variation unique to each SES dimensions (Supplementary Fig. 10).

To annotate the delineated relationship between the brain imprint of SES and mental processes, we have built on the Neurosynth database. We obtained probabilistic measures for the degree to which specific terms (such as “attention,” “emotion,” and “sleep”) are functionally linked to specific brain regions (Yarkoni et al. 2011). These ensuing quantities reflect how often specific ontological terms and brain locations have co-occurred in thousands of published research articles. We hence assessed the correlation of the SES-related brain patterns against the individual term maps from this database (Fig. 5).

Across the interrogated 6 SES indices, functional annotations from Neurosynth with large positive loadings were related to mental skills or skills development (e.g. “consolidation,” “expertise,” “mental imagery,” “rehearsal,” or “strength”). Negative term associations included attention- and working-memory-related capacities (e.g. “visual attention” and “focus”), but also management of interpersonal relationships (e.g. “communication,” “competition,” “empathy,” and “facial expression”). We made similar observations and conclusions when computing the functional associations separately for each brain hemisphere (Supplementary Fig. 9). We also provide results after an additional partial regression step to elucidate that meta-analytic associations that are uniquely or specifically explained by 1 of the 6 SES measures (Supplementary Fig. 10). Taken together, SES-related patterns of gray and white matter were associated with behavioral functions involving mental focus, social competence, and competitive drive.

## Discussion

In human societies, and those of other primates, individuals of low SES are more vulnerable to mental and physical disease (Sareen et al. 2011; Stringhini et al. 2017). Individuals of high SES and of low SES are known to diverge in their behavioral repertoire and daily lifestyle choices. Such predispositions and tendencies have deep consequence for well-being and life trajectories (Denney et al. 2014). The present study focused on the ≈10,000 UK Biobank release to carefully delineate the relationship between socioeconomic position and human behavior. By developing a multivariate multi-output pattern-learning framework, we have detailed how key SES indicators are reflected in brain architecture, as measured by variation in regional brain volume and fiber tract microstructure. The core insight from our analysis is that SES aspects are reflected in both gray matter volume and white matter integrity in a diametrically opposite way in both brain hemispheres.

Broadly, a convergent spatial distribution of asymmetry effects emerged across all the identified SES manifestations in the brain. It is important to keep in mind that SES is not a monolithic term in our study. The brain-SES associations revealed a global pattern of left–right asymmetry that became consistently apparent for each of the examined 6 SES dimensions. Individuals in the upper SES echelons tended to have larger brain volume effects in several regions of the left brain and smaller volume effects in several regions of the right brain; vice versa for individuals of low SES. This observation of left–right divergence in brain substrates explaining SES also became apparent for subcortical brain regions for most examined SES dimensions. In our white matter analyses, we made the analogous observation for the microstructure of anatomical fiber tracts. That is, individuals with higher socioeconomic standing tended to showed stronger effects in microstructure measures in fiber tracts in the left brain, whose homologues in the right brain were weaker, and vice versa.

In contrast, the handful of existing brain-imaging studies on SES have typically limited attention to the frontal lobe (e.g. Tomarken et al. 2004; D'Angiulli et al. 2012; Vargas et al. 2020). In these earlier studies, left hemisphere differences were repeatedly discussed in the context of language and semantics capacities. However, some brain-imaging studies showed that left-hemisphere effects in SES remained significant after controlling for language test performance (Raizada et al. 2008). Such residual effects were for instance shown in the left inferior frontal gyrus including Broca’s area. Instead, right hemisphere differences in SES studies were frequently discussed in the context of immunosuppression, cortisol levels, neighborhood deprivation, as well as stress exposure and health disparity (Lewis et al. 2007; D'Angiulli et al. 2012; Hanson et al. 2012; Vargas et al. 2020).

The here-discovered brain signature is consistent with the interpretation of a reduced *degree* of brain lateralization in individuals placed in the lower layers of society. This speculation is motivated from reanalysis of neuropsychological tasks (Boles 2011). This study confirmed that behavioral differences between individuals of high and low SES are consistent with prominence vs. paucity of hemispherically differentiated task responses. The investigators revealed a consistent relationship between SES and hemispheric asymmetry as measured by lateral differences in dichotic listening, tactile dot enumeration, visual emotion, and word recognition. The findings were interpreted by the author as being consistent with maturation delay or reduced hemispheric specialization in groups of lower SES (Boles 2011).

Given the copious sample size (*n* ≈ 10,000) in the present investigation, our findings can be expected to be fairly robust. A previous meta-analysis of 11 small-sample studies, each with less than 300 participants, indicated that high SES, as compared with low SES, is linked to decreased volume of the left superior frontal cortex and orbital frontal cortex (Yaple and Yu 2020). The authors also linked high SES to increased gray matter volume of left and right hippocampus as well as of right precuneus. Another large-sample study (*n* = 1099) has associated higher parental education (as an indicator of higher SES) with increased hippocampal volume in children and adolescents (Noble et al. 2015). In addition, this population study reported that family income specifically accounted for significant variation in surface area in the bilateral inferior frontal, cingulate, insula, and inferior temporal regions, as well as in the right superior frontal and precuneus cortex. Several of these reported regions are implicated in language-related processing and executive functioning (Noble et al. 2015). These early hints from previous SES studies are mostly based on single parental or subjective SES measures. This circumstance may explain some discrepancy from our own results. These findings, however, overlap with our results in that we confirm the left hippocampus to be associated with income. Moreover, our findings are in line with other previous reports that have found a relationship of SES with the microstructure of the fornix and cingulum bundle (Ursache et al. 2016; Dufford and Kim 2017). Extending this prior work, our multi-trait and multi-tissue analyses confirmed SES-sensitive features of brain architecture and extended them to the population level.

More specifically, anatomical variation in the caudate nucleus and temporal pole regions and in the posterior thalamic radiation tract here showed a positive relation to 2 of the SES dimensions education degree and neighborhood level. An exhaustive review of cognitive functions supported by the caudate nucleus concluded (Grahn et al. 2008) that this brain region is implicated in exciting correct action schemas and in selecting appropriate sub-goals based on an evaluation of action-outcomes, in addition to its commonly mentioned role in motor function. This SES-related region may be involved in neurocognitive processes that are fundamental to the successful pursuit of short-term tactics and long-term strategies (Grahn et al. 2008).

Additionally, neurological lesion studies and neuropsychological studies in bilingual individuals also point to the preferential implication of the left caudate nucleus in the neural mechanisms underlying language selection and control (Caplan et al. 1990; Zou et al. 2012). Much of human education is intimately related to semantic concepts and word choice and may thus explain a part of our brain-SES associations. The left temporal pole, here significantly associated with higher degrees and having a knowledge worker (“white-collar”) job, is similarly instrumental for realizing language capacities. This SES-sensitive brain region is widely considered to be a functional hub for semantics and concept comprehension (Tsapkini et al. 2011). A review of the literature on both nonhuman and human primates extends this suggested role of the temporal pole to higher processes necessary for social interaction. Such high-level functional involvements go beyond semantic memory and imply relevance of personal semantic memory by storage of perception-emotion links (Olson et al. 2007), abstract cultural knowledge, and “scripts” of adequate social behavior for the variety of contexts of everyday life (Zahn et al. 2007). Taken together, the socioeconomic position of individuals is related to interindividual anatomical variation in brain features that have been linked to neurocognitive processes, which underlie goal-directed behavior, language capital, and rules that provide a scaffold for everyday social interplay.

Our meta-analytic query of the Neurosynth database has linked the collective gray matter substrates of SES to functional aspects of mental focus, social competence, and competitive drive. The delineated structure–function annotations substantiate earlier studies arguing that SES differences mediate hemispheric asymmetry in a way that impacts attentional performance and dealing with motivational states (D'Angiulli et al. 2012). Such cerebral asymmetry, especially in the frontal lobe, can be attenuated by acute and accumulated life stress, such as due to traumatic experience (Carrion et al. 2001; Lewis et al. 2007; Hanson et al. 2012). The size of this attenuation effect was found to be tied to poor health status (Lewis et al. 2007), which is known to be linked to low SES. Specifically, variation in structural and functional brain asymmetry was thought to be driven by early childhood stress (Carrion et al. 2001; Hanson et al. 2012). The conditions of early childhood experience are itself influenced by socioeconomic conditions and mood disorders of caretakers (Tomarken et al. 2004). Again, a wide ranging restriction to the frontal lobe characterizes many of these previously published accounts on SES and brain lateralization. Our spatially impartial study extends these previous cues in the neuroscience literature by showing that hemispheric asymmetry represents an principled brain pattern with an intimate relationship to what underpins success and status in society. Future brain-imaging studies could take a step forward and investigate more specifically to what extent hemispheric asymmetry is associated with the degree of mental focus, social competence, and competitive drive.

More broadly, as one previously proposed explanation, hemispheric specialization might have been spurred by hemispheric conduction delay (Ringo et al. 1994). Several investigators have put forward that during evolution, brain asymmetries may have developed as a biological adaptation related to more efficient processing of information (Corballis 2017). It has been argued that aligning the direction of behavioral asymmetries in a population may have arisen as an ‘evolutionarily stable strategy’ under social selection pressures. This scenario could have occurred when individual behavior needed to be coordinated with the behavior of other organisms, with asymmetrical specialization, of the same or different species (Vallortigara and Rogers 2005): “Brain and behavioral lateralization, as we know it in humans and other vertebrates, may have evolved under basically ‘social’ selection pressures” (Ghirlanda and Vallortigara 2004). Additionally, brain asymmetry in humans has been reported to be more variable than in apes (Neubauer et al. 2020). This finding may reflect increased anatomical compartmentalization and functional modularization of the human brain. More nuanced segregation of functional brain organization could provide a scaffold for a richer and more sophisticated behavioral repertoire.

In the light of present and previous research, there may be evolutionary advantages to increasingly sophisticated hemispheric specialization (Hartwigsen et al. 2021), which may resurface as special relationships in how the left and brain hemisphere are associations with SES dimensions in the present study. However, there may also be environmental influences correlated with SES, which in turn factor into the development of hemispheric specialization. Our findings would be consistent with the idea that hemispheric asymmetry is related to SES because the degree of hemispheric specialization has been associated with better mental performance—the extent of hemispheric asymmetry is probably an important ingredient that distinguishes the human brain from that of other animals (Hartwigsen et al. 2021). These collective findings support the notion that brain asymmetry may link to the aspects of socioeconomic disadvantages or socioeconomic privilege.

In conclusion, by repurposing algorithmic tools for mining the UKBB resource, we began to reveal the characteristic brain-global imprint of SES in the wider society. As the central conclusion from our structural brain-imaging study, an individual’s socioeconomic position may resonate preferentially with patterns of brain lateralization between both hemispheres. Several brain regions and fiber tracts were robustly associated with SES indicators in the positive direction in the left brain and in the negative direction in the right brain. This population-level insight paves the way for future investigations into the interplay of SES with the neural circuits that govern human behavioral tendencies, daily choices, and their long-term sequelae for health outcomes. Such studies should employ a longitudinal design to scrutinize the causal influence that SES exerts on brain structure and function.

## Funding

This project has been made possible by the Brain Canada Foundation, through the Canada Brain Research Fund, with the financial support of Health Canada; National Institutes of Health (NIH R01 AG068563A, NIH R01 R01DA053301-01A1); the Canadian Institute of Health Research (CIHR 438531, CIHR 470425); Healthy Brains Healthy Lives initiative (Canada First Research Excellence fund) by the CIFAR Artificial Intelligence Chairs program (Canada Institute for Advanced Research) and by Google to DB.

*Conflict of interest statement:* The authors declare that they have no competing interest.

## Authors’ contributions

TBP and DB contributed to conception and design of the work. All authors contributed to interpretation of the data as well as writing the manuscript. DB led data analysis.

## Data availability

All data necessary to repeat the reported analyses and assess the conclusions are openly available by the UK Biobank (http://www.ukbiobank.ac.uk).

## Code availability

The analysis scripts that reproduce the results of the present study are available upon request.

## Supplementary Material

SES_Draft10_for_CCC_final_SOM_tgac020
