# Supplementary material for: Pattern learning reveals brain asymmetry to be linked to socioeconomic status
Source: Cereb Cortex Commun. 2022 May 20;3(2):tgac020. doi: 10.1093/texcom/tgac020 (PMC9188625; doi:10.1093/texcom/tgac020)
Supplement: SES_Draft10_for_CCC_final_SOM_tgac020 [file ses_draft10_for_ccc_final_som_tgac020.docx]

**Supplementary Online Material**

**Supplementary Material and Methods**

*Comparing different classes of machine learning algorithms for model selection*

In a preparatory set of analyses, we have put to the test a variety of commonly used pattern-learning algorithms to predict SES. The goal of this initial step of our investigation was to identify the modeling framework that achieves the best prediction performance of interindividual differences in SES based on brain patterns in new participants (i.e., out-of-sample prediction accuracy).

Linear models were represented by multi-output ridge regression (default choice of alpha=0.01). Non-linear models based on ensembles of decision trees were represented by random forest algorithms (hyper-parameters: number of trees=100, 200, 300, max depth=2, 4, 6). Non-linear models based on forward stagewise fitting of a sequence of nested estimators were represented by the gradient boosting algorithm (hyper-parameters: estimators=100, 200, 300, max depth=2, 4, 6). Non-linear models using fully non-parametric estimation without assuming a specific functional form were represented by k-nearest neighbor algorithms (hyper-parameters: number of neighbors=1, 2, …, 25). Non-linear models involving implicit expansion of the original feature space into a vast unbounded alternative space were represented by kernel-augmented ridge regression (hyper-parameters: alpha=-4 to +4 in 9 steps in logarithmic space, gamma=-10 to 0 in 11 steps in logarithmic space). Non-linear models based on learning hierarchically non-linear representations involving rectified-linear units (ReLUs) for adaptive basis-function regression were represented by deep neural network algorithms (hyper-parameters: hidden layers=10, 20-10, 50-20-10, learning rate=0.01, 0.001, 0.0001, default solver=ADAM, default weight decay penalization alpha=0.0001, default batch size=200 data points, default Nesterov moment=turned on).

To safeguard against the risk of overfitting, each of these candidate machine learning algorithms was assessed by means of a rigorous nested cross-validation scheme. For each algorithm, in each of 10 fit-tune-predict cycles, the total number of (original) 10,000 UK Biobank participants was divided into 90% training subset and 10% testing subset (10-fold outer cross-validation). The adjustable parameters of the learning model were first fitted on the training subset of participants, and the built model was then evaluated on the unseen, independent participants of the 10% testing subset. For the purpose of hyper-parameter tuning the training subset was itself divided into 3 portions (3-fold inner cross-validation): the first two thirds used for model fitting of each combination of hyper-parameters, and the remaining third portion (of the outer training subset) to pick the best among the competing hyper-parameter combinations. The ensuing 10 out-of-sample prediction estimates were then averaged across the 10 outer test subsets to obtain a single cross-validation estimate of the expected prediction performance in future data.

To acknowledge idiosyncrasies or subjectivity of any single choice of performance criterion, we have carefully indexed the error rates based on five complementary metrics of prediction accuracy on the unseen test participants (Hastie et al. 2015; Goodfellow et al. 2016): 1) Pearson’s correlation coefficient (r^2^), 2) explained variance score (EVS), 3) mean absolute error (MAE), 4) coefficient of determination (COD), and 5) mean squares error (MSE). Based on these systematic explorations of the model design space, our collective result have indicated multi-output ridge regression to achieve the overall most convincing performance (Table 2 and 3 chart out-of-sample results; Supplementary Tables 12 and 13 chart in-sample results). In particular, the simple linear model was not clearly outperformed by any of the non-linear high-capacity algorithms that we have submitted to the direct model comparison across several different performance metrics (cf. He et al. 2020; Kiesow et al. 2020; Schulz et al. 2020).

**Supplementary References**

Goodfellow IJ, Bengio Y, Courville A. 2016. Deep learning. USA: MIT Press.

Grahn JA, Parkinson JA, Owen AM. 2008. The cognitive functions of the caudate nucleus. Prog Neurobiol. 86:141-155.

Hastie DI, Liverani S, Richardson S. 2015. Sampling from Dirichlet process mixture models with unknown concentration parameter: mixing issues in large data implementations. Stat Comput. 25:1023-1037.

He T, Kong R, Holmes AJ, Nguyen M, Sabuncu MR, Eickhoff SB, Bzdok D, Feng J, Yeo BT. 2020. Deep neural networks and kernel regression achieve comparable accuracies for functional connectivity prediction of behavior and demographics. NeuroImage. 206:116276.

Kiesow H DR, Kable JW, Kalenscher T, Vogeley K, Schilbach L, Marquand A, Wiecki TV, Bzdok D. 2020. 10,000 Social Brains: Sex Differentiation in Human Brain Anatomy. Science Advances.

Schulz M-A, Yeo BT, Vogelstein JT, Mourao-Miranada J, Kather JN, Kording K, Richards B, Bzdok D. 2020. Different scaling of linear models and deep learning in UKBiobank brain images versus machine-learning datasets. Nature communications. 11:1-15.

**Supplementary Figure 1**


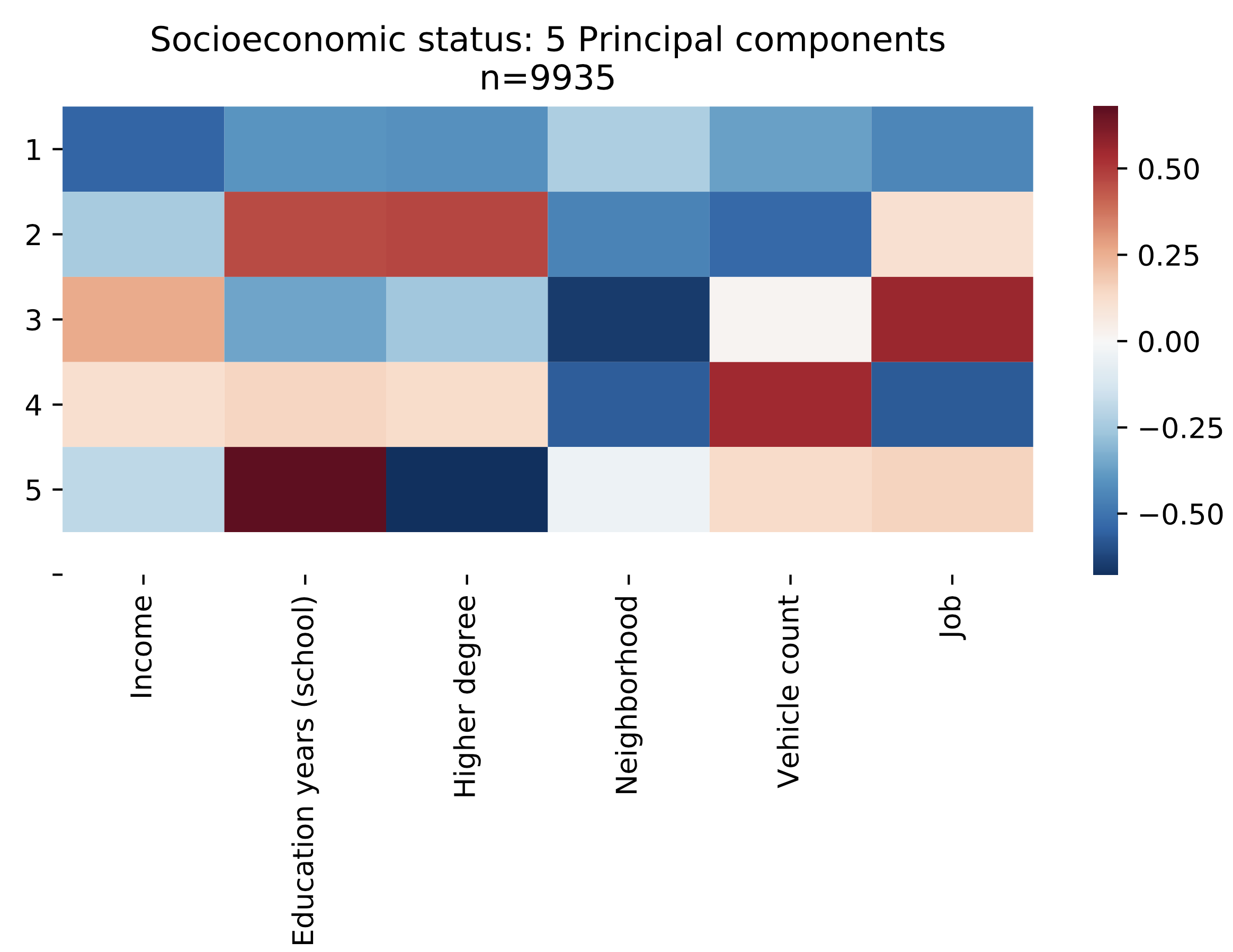


**Mixed-membership decomposition of socioeconomic status (SES) dimensions.** As a preliminary data exploration step, principal component analysis demonstrated useful combinations of socioeconomic indices for decomposing the construct SES. The overall variance was mainly explained by distinct latent principal dimensions of variation that involve particular single SES indicators. The color bar represents the dimension-wise loadings from the extracted principal components. The explained variance from principal component 1 to 5 are 0.32, 0.22, 0.15, 0.12, and 0.10, respectively.

**Supplementary Figure 2**


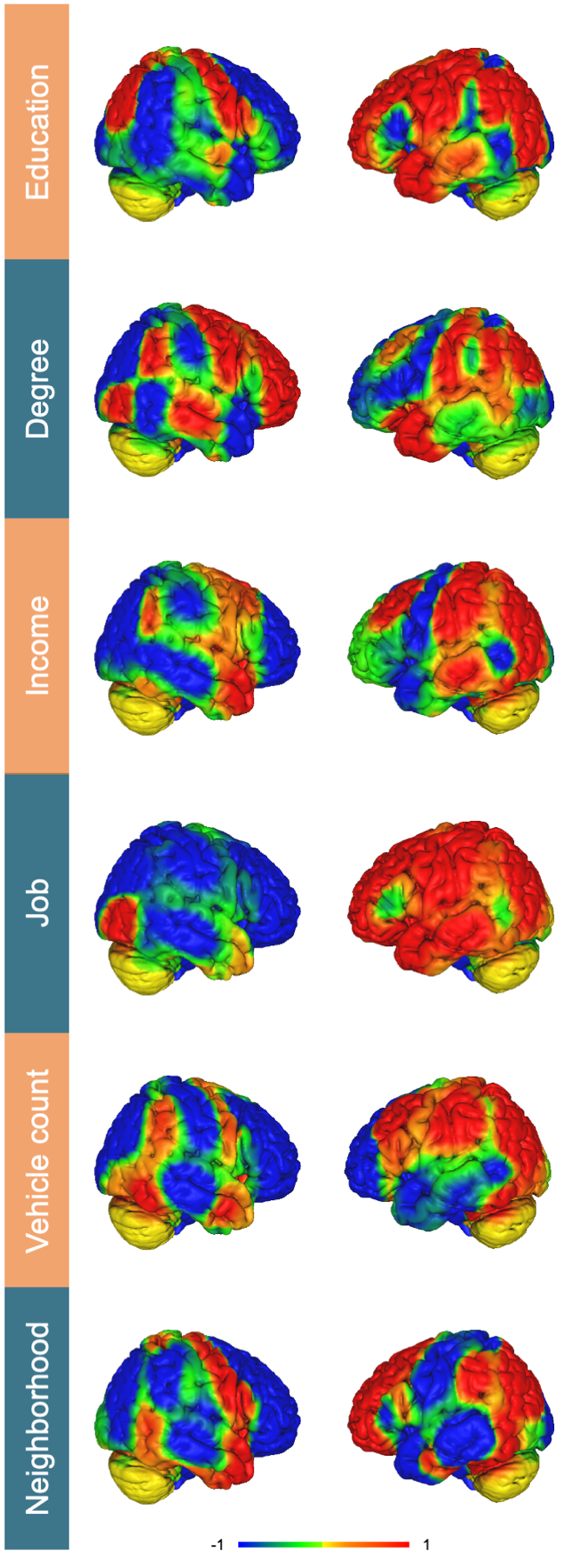


**Cortical effect sizes of brain-SES associations.** Shows full parameters from the multi-output model without thresholding or significance testing (cf., Supplementary Table 2). These estimated model parameters are shown on z-scale (i.e., mean-centered and unit-variance scaled) for the sake of comparability across the 6 SES dimensions. Hence, -1 or +1 indicate a fitted model parameter that is 1 standard deviation smaller or larger than the average of parameters in the model.

**Supplementary Figure 3**

**
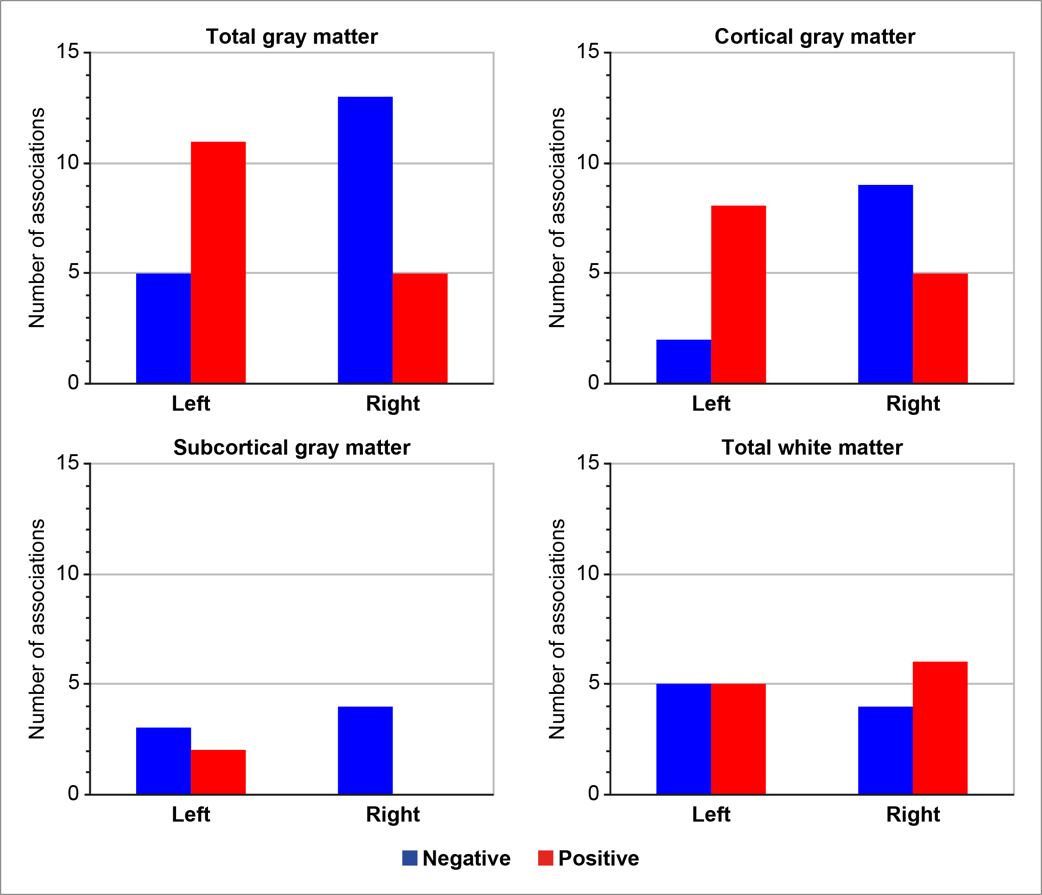
**

**Hemispheric lateralization patterns of socioeconomic status-brain associations.** Hemisphere-wise comparison of the directions of socioeconomic status-brain associations revealed a hemispheric asymmetry of gray matter relevances that was driven by cortical regions. As an overarching pattern, associations were predominantly positive in the left hemisphere, whereas negative associations were predominantly found in the right hemisphere.

**Supplementary Figure 4: Replication of main findings in 3 separate samples of 10,000 UK Biobank participants in structural MRI**

**
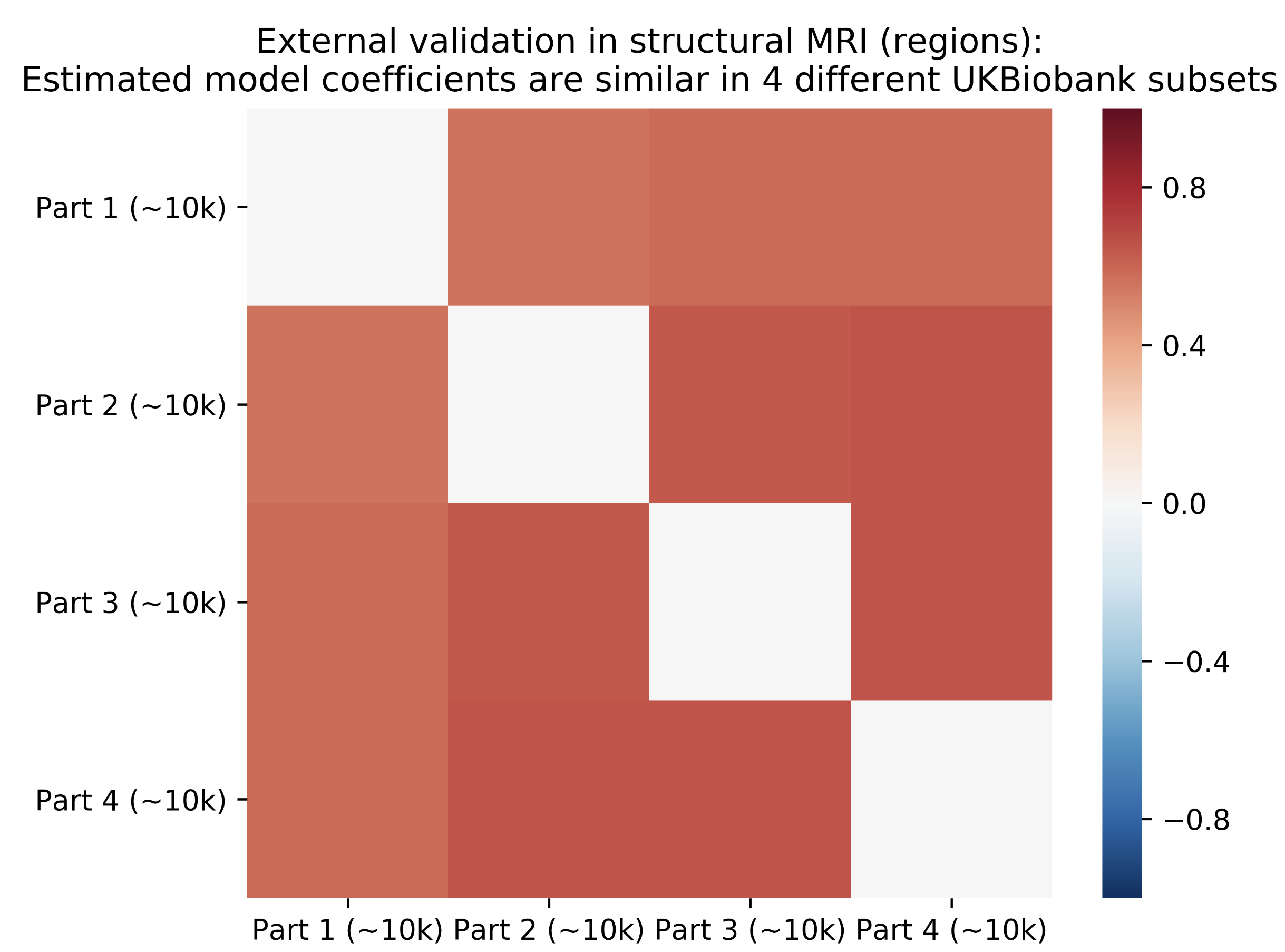
**

**Supplementary Figure 5: Replication of main findings in 3 separate samples of 10,000 UK Biobank participants in diffusion MRI**


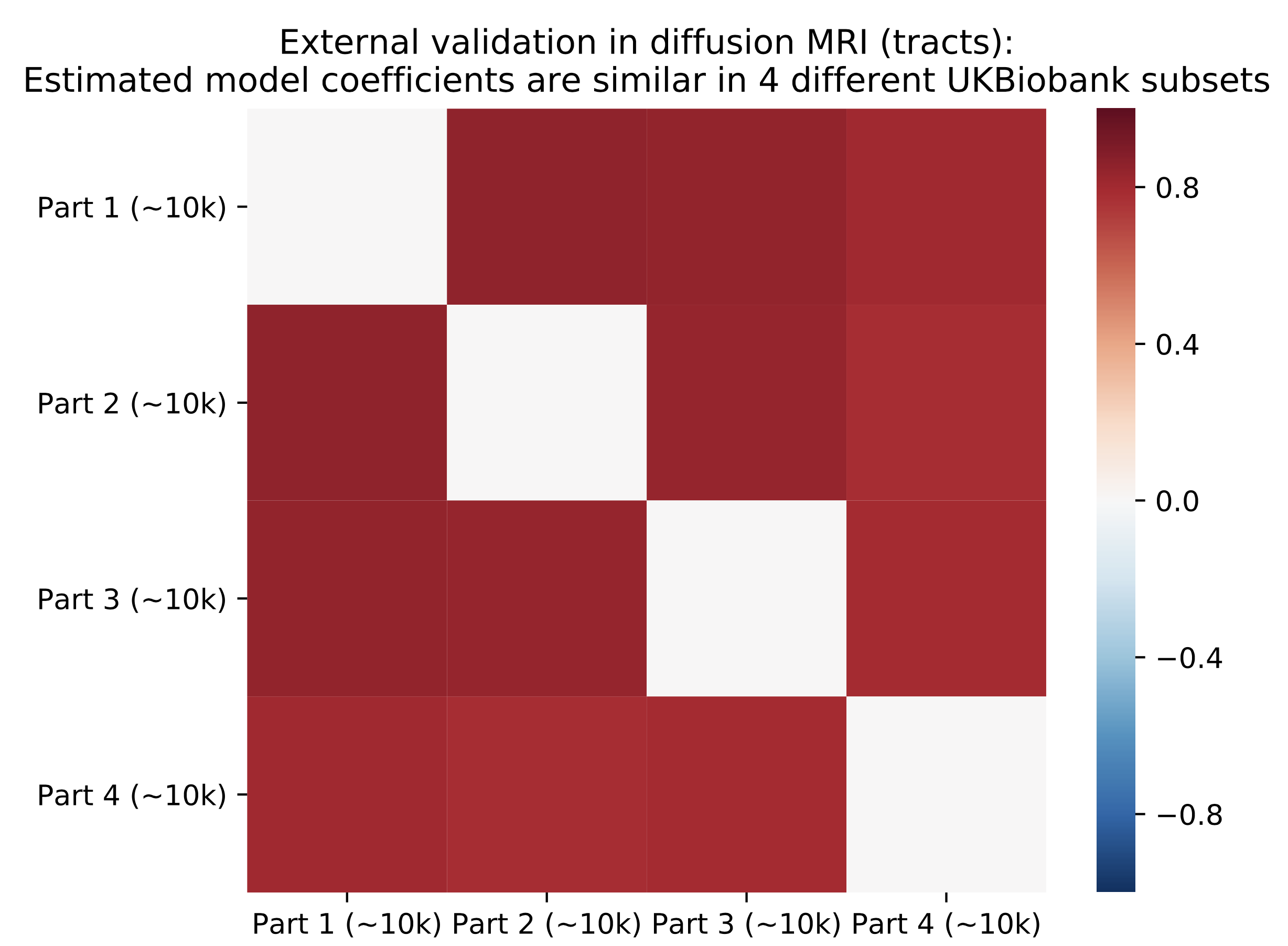


**Supplementary Figure 6: Replication 1 of main findings in additional 10,000 UK Biobank participants**


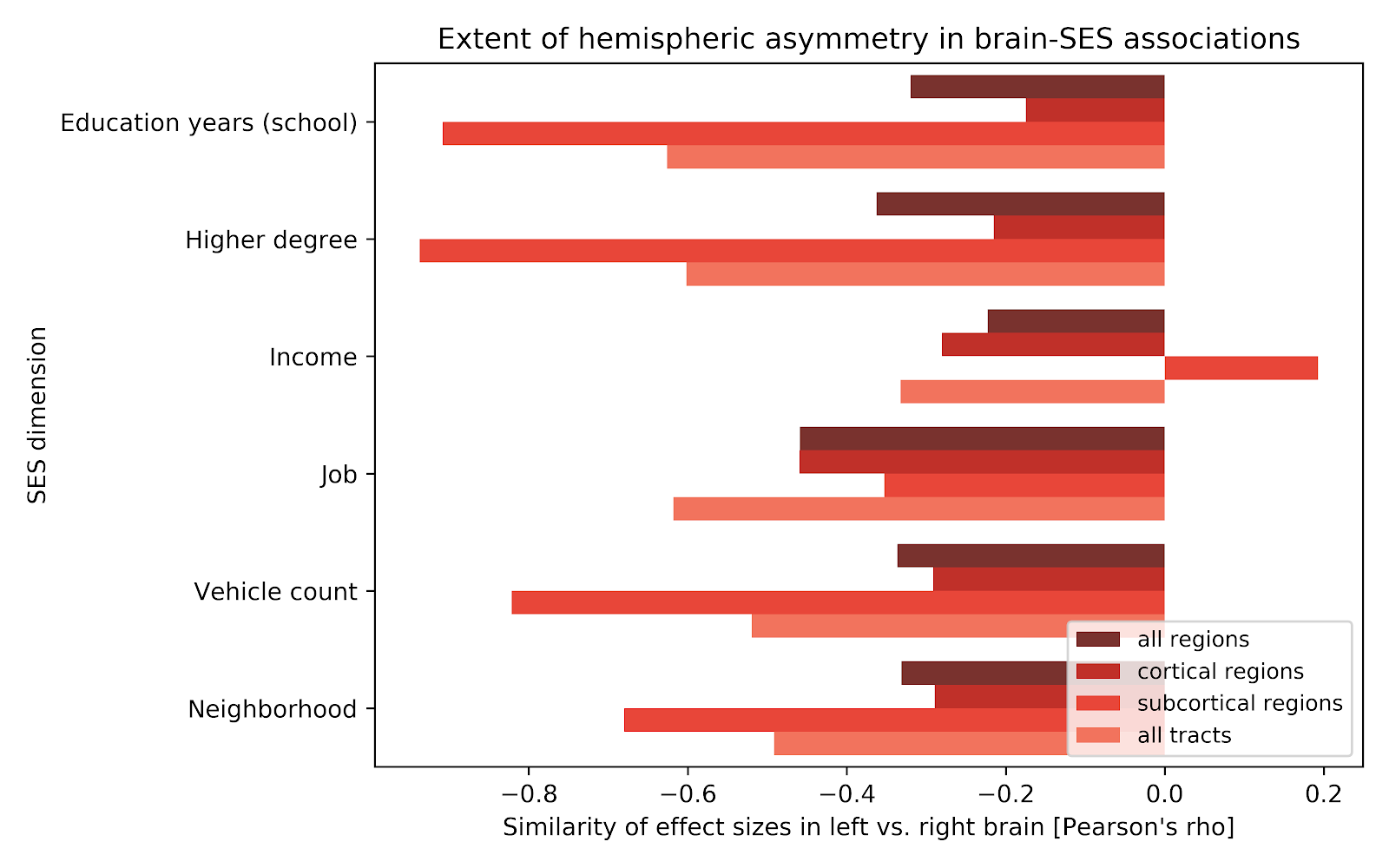


**Supplementary Figure 7: Replication 2 of main findings in additional 10,000 UK Biobank participants**


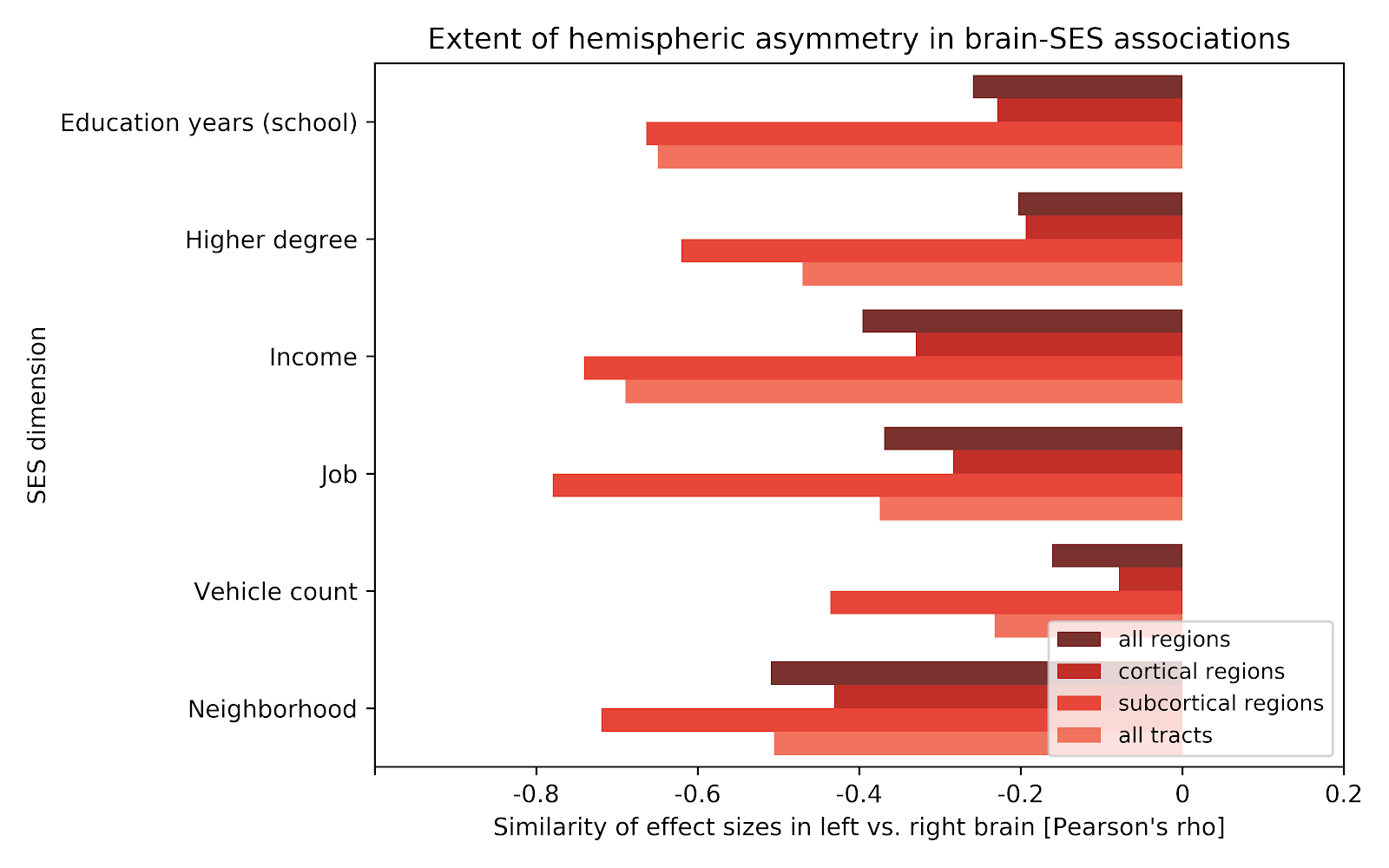


**Supplementary Figure 8: Replication 3 of main findings in additional 10,000 UK Biobank participants**


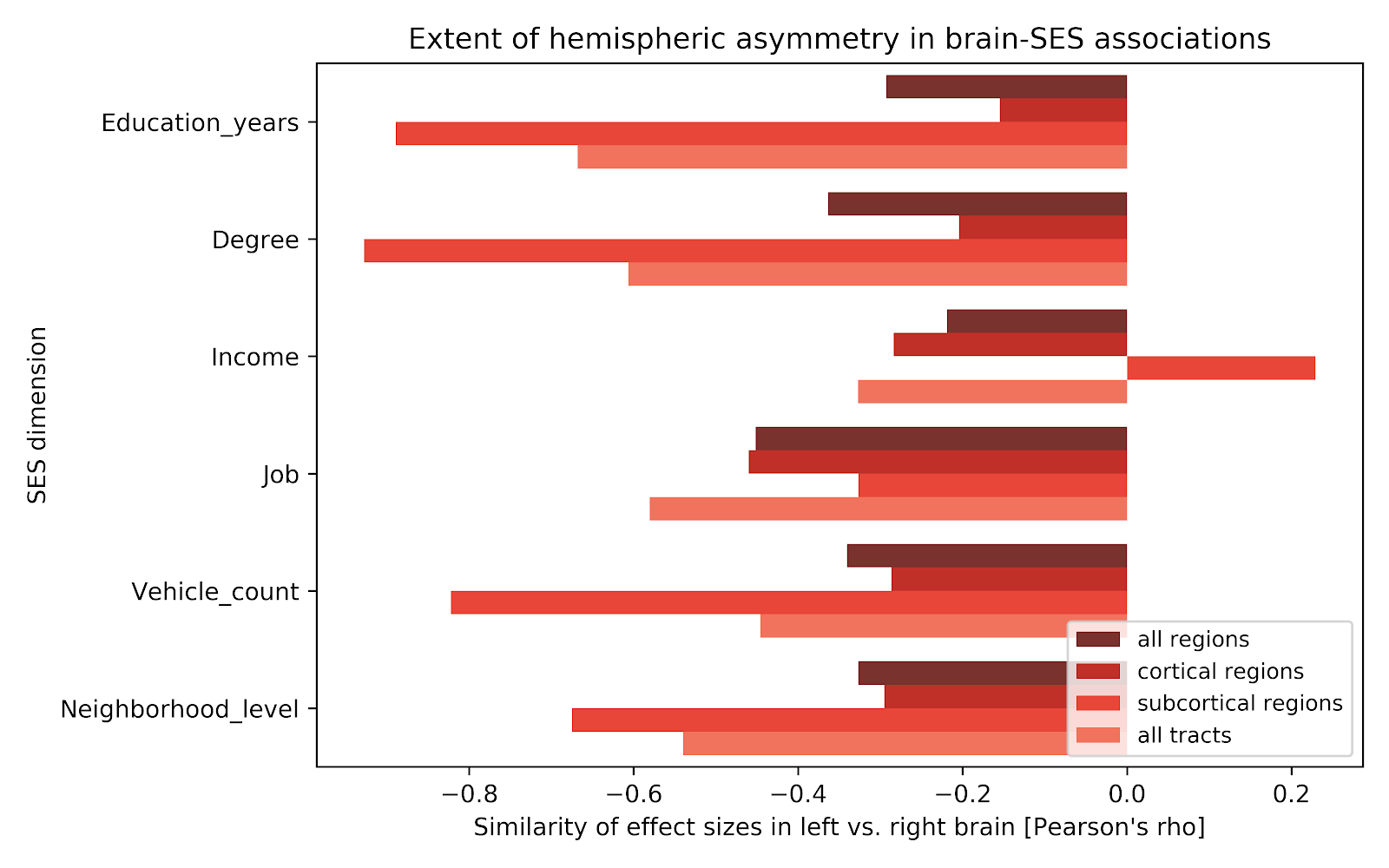


**Supplementary Figure 9**


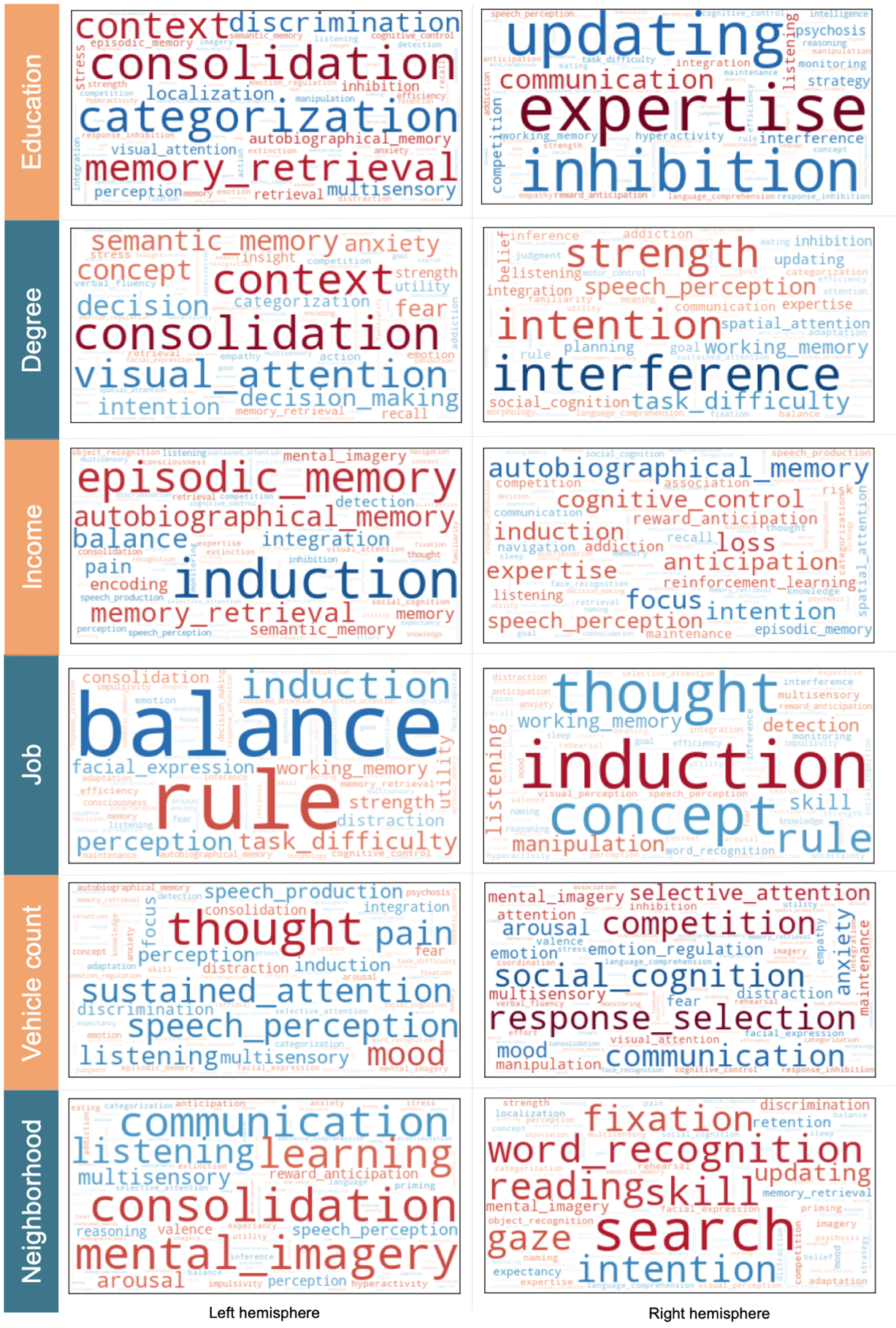


**Functional annotation of how socioeconomic status (SES) is linked to measures of brain architecture in the left vs. right hemisphere.** Neurosynth, a large-scale database of brain-imaging experiments was queried for co-occurrence with ontological terms that map onto the derived gray-matter-wide patterns of SES associations. We computed the similarity between a given term’s functional activity patterns and the obtained brain correlates of SES (*red/blue* = positive/negative correlation, range = [-0.3, 0.3]) separately for the left and the right hemisphere. Word size represents the relative magnitude of a given association.

**Supplementary Figure 10**

**
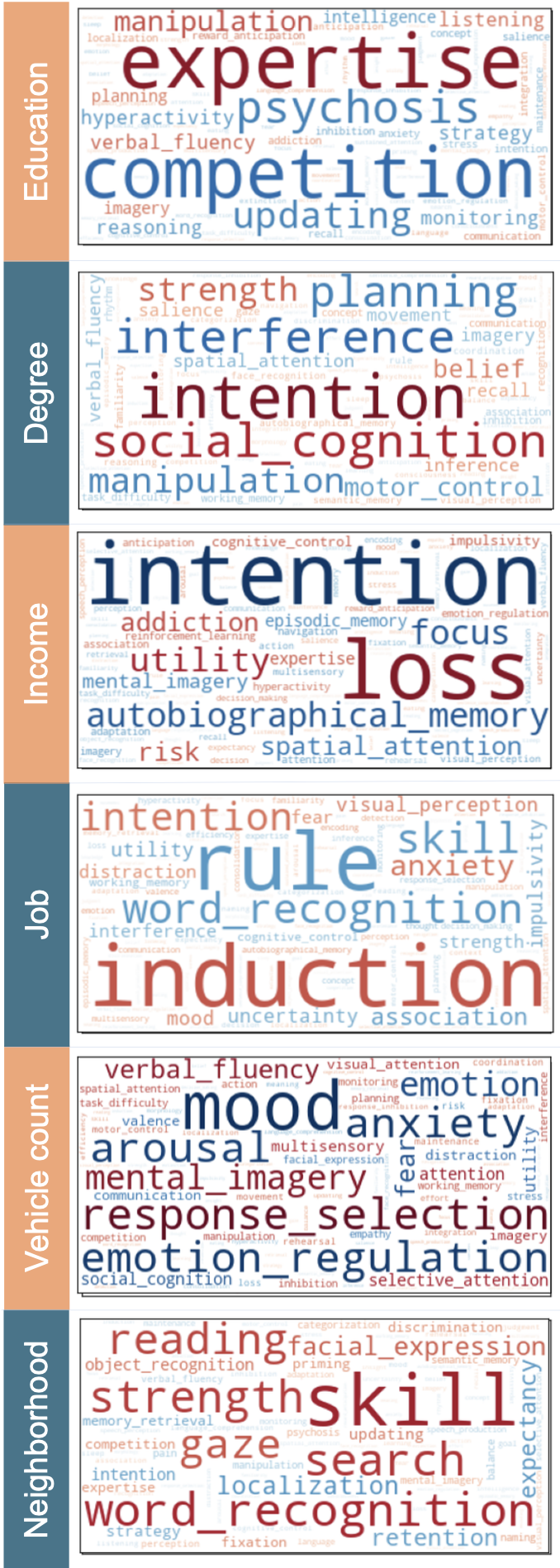
**

**Specific functional annotation of how socioeconomic status (SES) is linked to measures of brain architecture in the left vs. right hemisphere.** Same meta-analytic profiling of SES correlates as in Figure 5 and SFigure 9, but joint variation between the SES indicators has been removed using partial regression.

**Supplementary Table 1**

|  | Percent | Mean | SD | Range |
| --- | --- | --- | --- | --- |
| Age |  | 55 | 7.5 | 40–70 |
| Sex  Female  Male | 52.4  47.6 |  |  |  |
| Ethnic background  British  Irish  Any other white background  Others  Household income (£)  31,000 to 51,999  52,000 to 100,000  18,000 to 30,999  Less than 18,000  Greater than 100,000  Age completed full time education  College/University degree  Neighborhood deprivation  Vehicles in household  Qualified job  Body mass index (BMI) | 91.7  2.7  2.3  3.3  27.7  22.6  21.7  12.4  5.4  44.4  49.5 | 17  1.9  2.7  26.7 | 2.3  2.7  0.8  4.3 | 5–35  -9.1–6.3  1–5  16.1–63.6 |

**Demographic information**

**Supplementary Table 2**

**
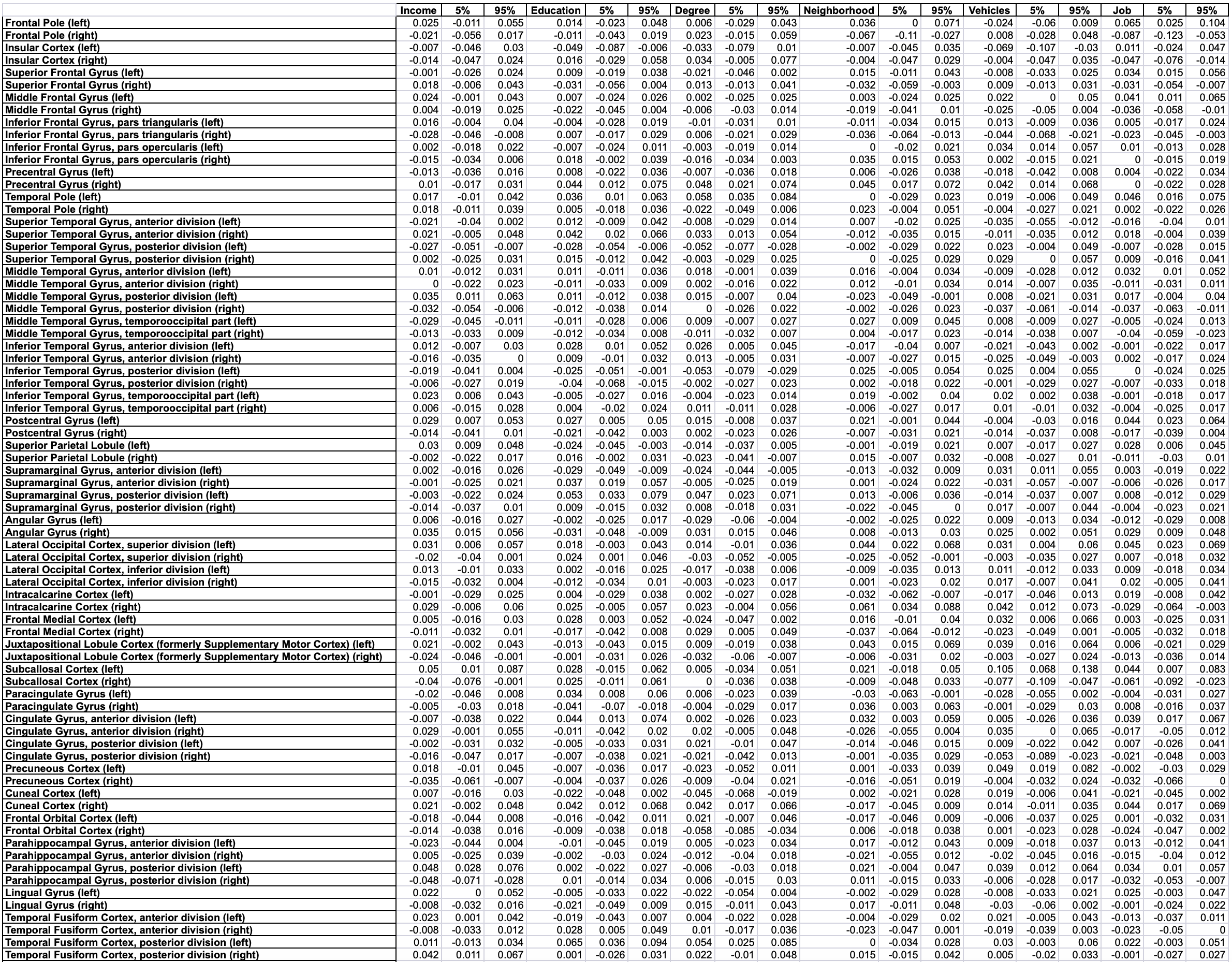
**

**
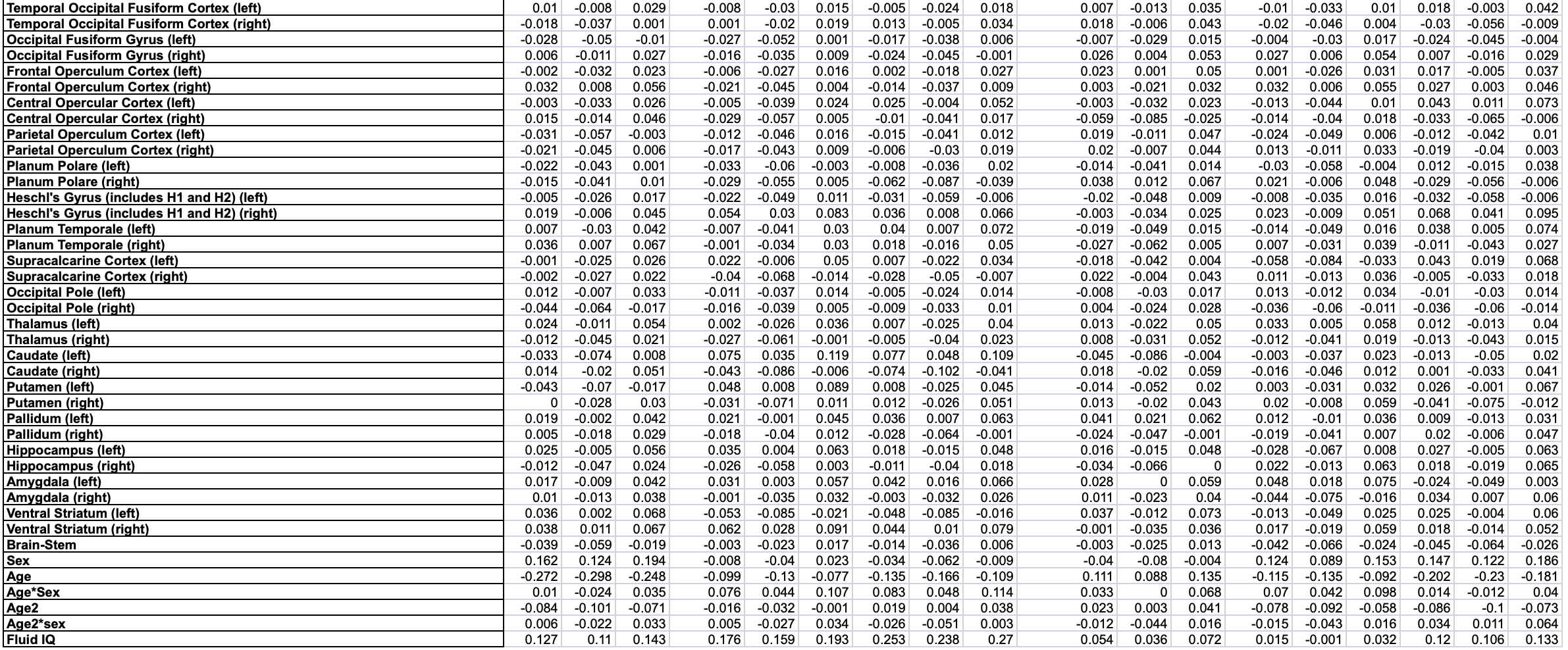
**

Effect sizes (parameter point estimate) and population confidence intervals (bootstrap-based uncertainty estimate) of gray matter associations with six socioeconomic status dimensions (with IQ covariate).

**Supplementary Table 3**

**
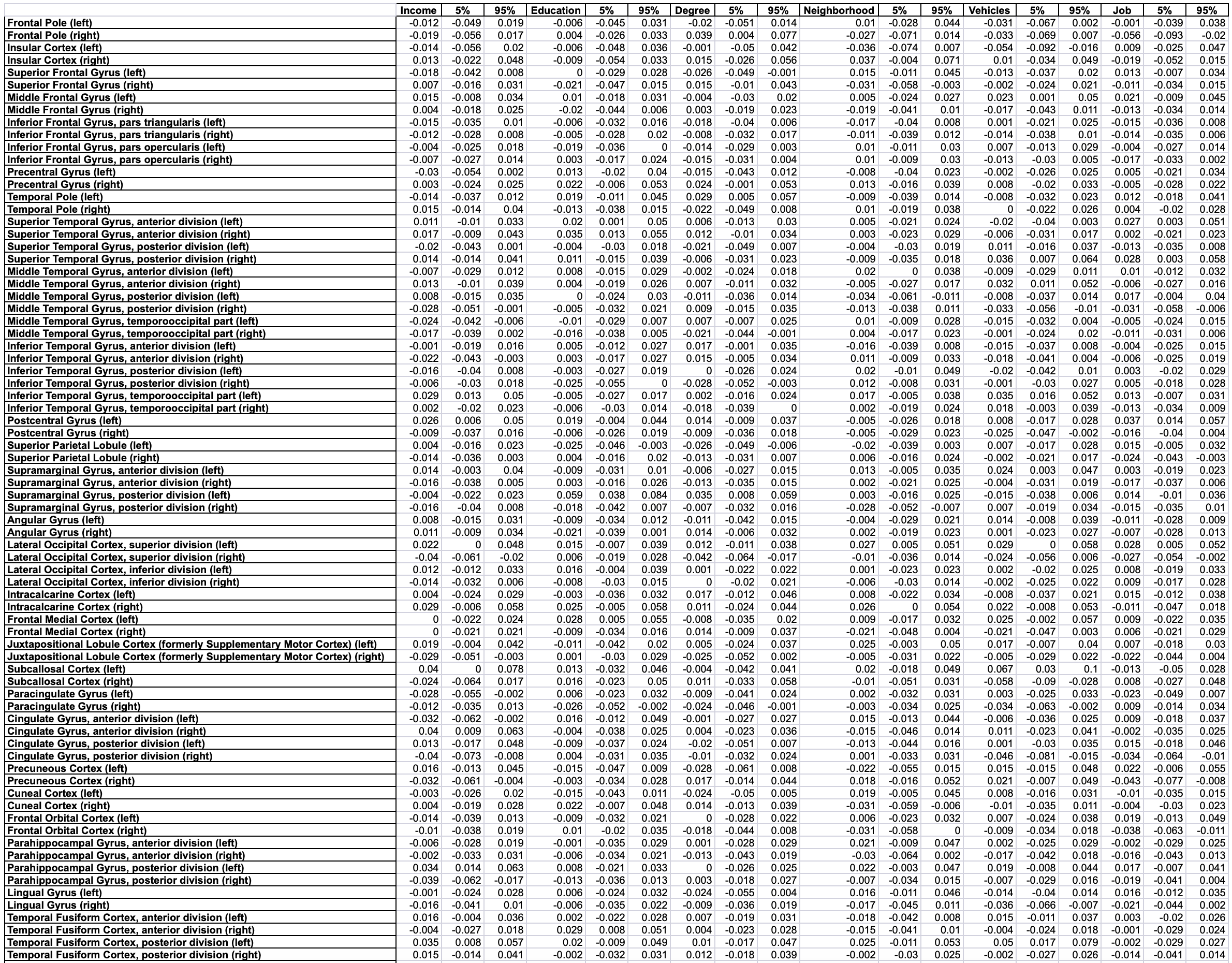
**

**
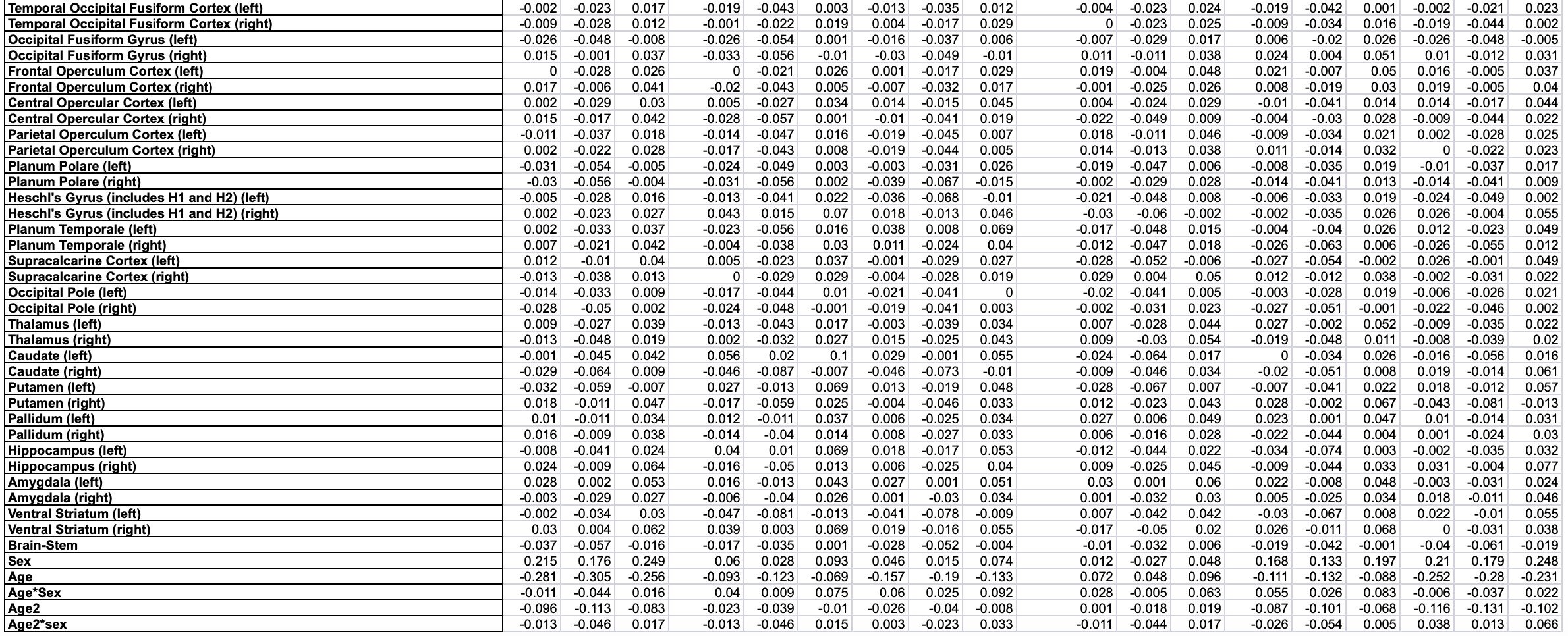
**

Effect sizes (parameter point estimate) and population confidence intervals (bootstrap-based uncertainty estimate) of gray matter associations with six socioeconomic status dimensions (without IQ covariate).

**Supplementary Table 4**

| **Region** | **Hemisphere and Direction** | | | |
| --- | --- | --- | --- | --- |
|  | **Left +** | **Left -** | **Right +** | **Right -** |
| **Brain-Stem** |  | 3 |  | 3 |
| **Caudate** | 2 |  |  | 1 |
| **Central Opercular Cortex** |  |  |  | 1 |
| **Cingulate Gyrus, posterior division** |  |  |  | 1 |
| **Cuneal Cortex** |  | 1 |  |  |
| **Frontal Orbital Cortex** |  |  |  | 1 |
| **Frontal Pole** | 1 |  |  | 1 |
| **Heschl's Gyrus (includes H1 and H2)** |  |  | 1 |  |
| **Inferior Frontal Gyrus, pars opercularis** |  |  | 1 |  |
| **Inferior Frontal Gyrus, pars triangularis** |  |  |  | 1 |
| **Inferior Temporal Gyrus, posterior division** |  | 1 |  |  |
| **Intracalcarine Cortex** |  |  | 1 |  |
| **Middle Temporal Gyrus, temporooccipital part** |  |  |  | 1 |
| **Parahippocampal Gyrus, posterior division** | 1 |  |  | 1 |
| **Planum Polare** |  |  |  | 1 |
| **Postcentral Gyrus** | 1 |  |  |  |
| **Precentral Gyrus** |  |  | 1 |  |
| **Subcallosal Cortex** | 1 |  |  | 1 |
| **Superior Temporal Gyrus, anterior division** |  |  | 1 |  |
| **Supramarginal Gyrus, posterior division** | 1 |  |  |  |
| **Temporal Fusiform Cortex, posterior division** | 1 |  |  |  |
| **Temporal Pole** | 2 |  |  |  |
| **Number of Associations** | **10** | **5** | **5** | **13** |

Spatial distribution of statistically significant brain-SES associations in gray matter. The first two rows specify anatomical nomenclature, hemisphere and direction of the association. Unpaired regions were attributed to each hemisphere, respectively.

**Supplementary Table 5**


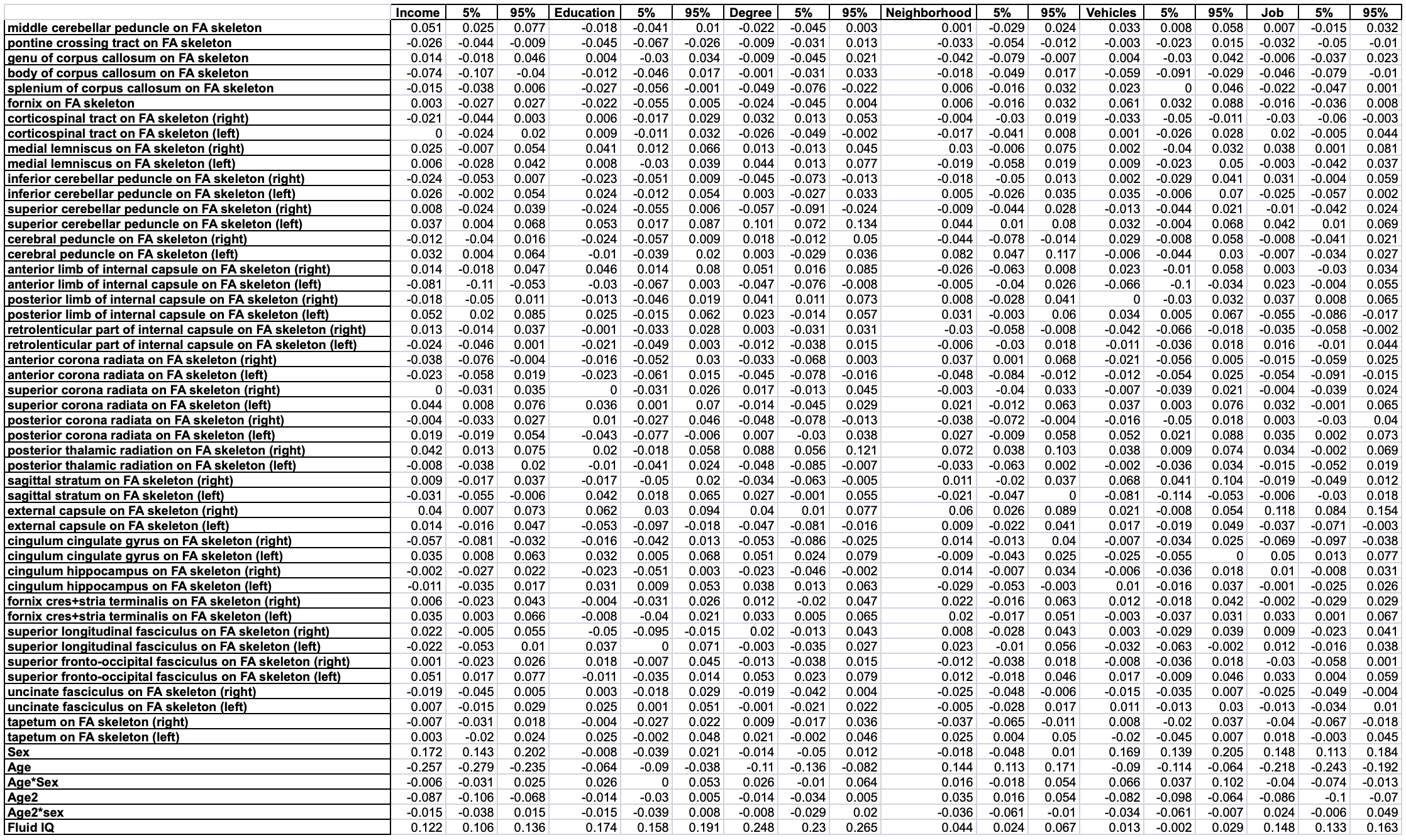


Effect sizes (parameter point estimate) and population confidence intervals (bootstrap-based uncertainty estimate) of white matter associations with six socioeconomic status dimensions (with IQ covariate).

**Supplementary Table 6**


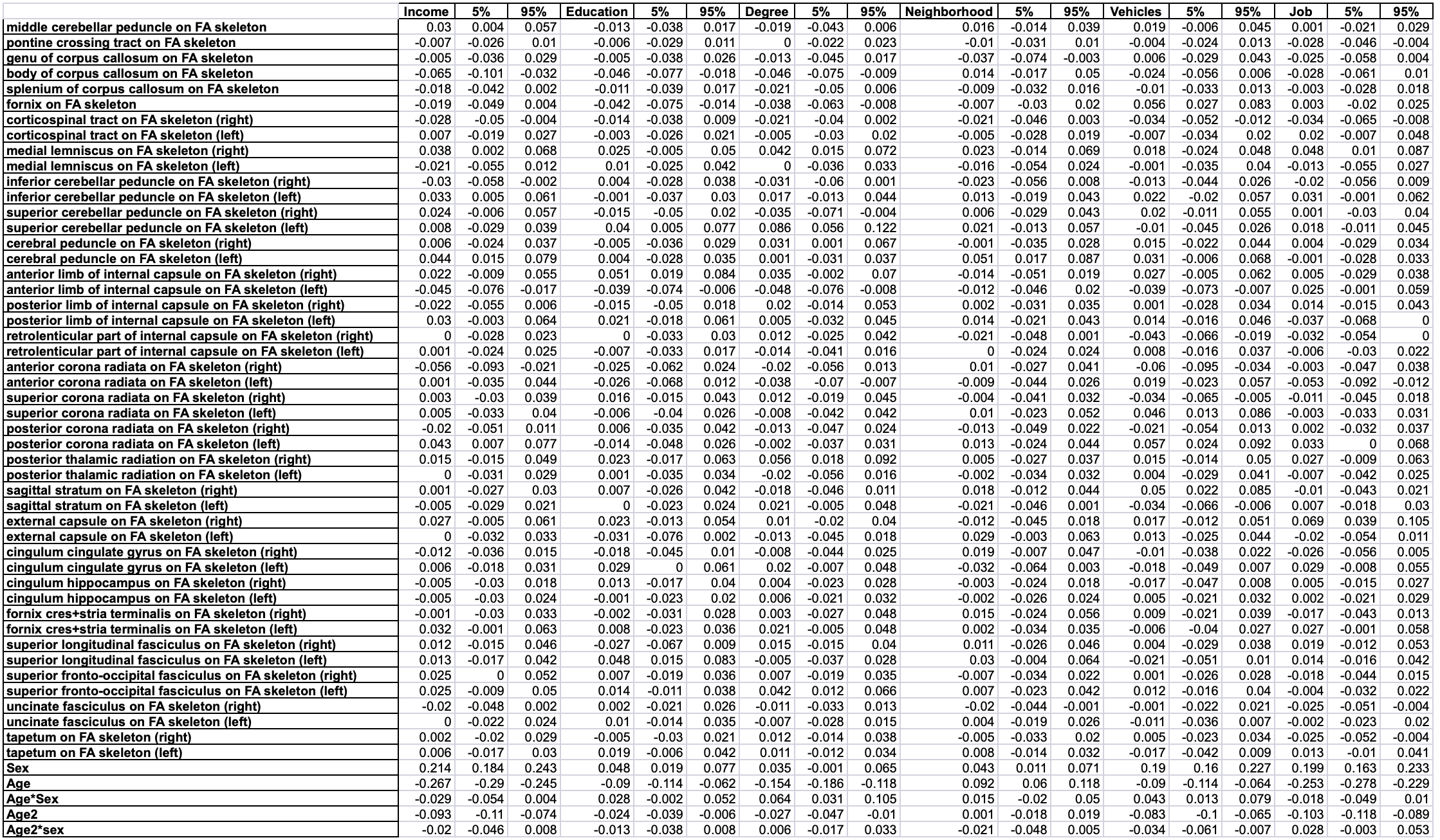


Effect sizes (parameter point estimate) and population confidence intervals (bootstrap-based uncertainty estimate) of white matter associations with six socioeconomic status dimensions (without IQ covariate).

**Supplementary Table 7**

| **Region** | **Hemisphere and Direction** | | | | |
| --- | --- | --- | --- | --- | --- |
|  | **Left +** | **Left -** | **Right +** | **Right -** | **Fiber Type** |
| **anterior limb of internal capsule on FA skeleton** |  | 2 |  |  | Projection |
| **body of corpus callosum on FA skeleton** |  | 1 |  | 1 | Commissure |
| **cerebral peduncle on FA skeleton** | 1 |  |  |  | Projection |
| **cingulum cingulate gyrus on FA skeleton** |  |  |  | 2 | Association |
| **external capsule on FA skeleton** |  |  | 1 |  | Projection |
| **fornix on FA skeleton** | 1 |  | 1 |  | Projection |
| **middle cerebellar peduncle on FA skeleton** | 1 |  | 1 |  | Projection |
| **pontine crossing tract on FA skeleton** |  | 1 |  | 1 | Projection |
| **posterior thalamic radiation on FA skeleton** |  |  | 2 |  | Projection |
| **sagittal stratum on FA skeleton** |  | 1 | 1 |  | Association |
| **superior cerebellar peduncle on FA skeleton** | 1 |  |  |  | Projection |
| **superior fronto-occipital fasciculus on FA skeleton** | 1 |  |  |  | Association |
| **Number of Associations** | **5** | **5** | **6** | **4** |  |

Spatial distribution of statistically significant brain-SES associations in white matter. The first two rows specify anatomical nomenclature, fiber type, hemisphere and direction of the association. Unpaired fiber tracts were attributed to each hemisphere, respectively.

**Supplementary Table 8: Region-by-region effects using standard linear regression in grey matter volumes.** Without shrinkage penalization and without multi-output modeling, we still find left-right divergences in brain-SES associations using Pearson’s correlation across effects of homologous regions from the left and right brain hemisphere.


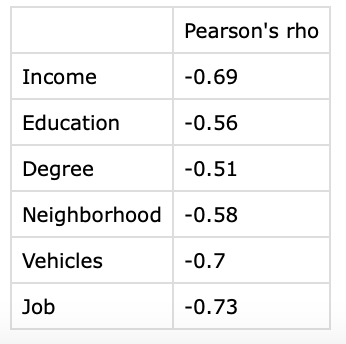


**Supplementary Table 9: Tract-by-tract effects using standard linear regression in white matter microstructure.** Without shrinkage penalization and without multi-output modeling, we still find left-right divergences in brain-SES associations using Pearson’s correlation across effects of homologous tracts from the left and right brain hemisphere.


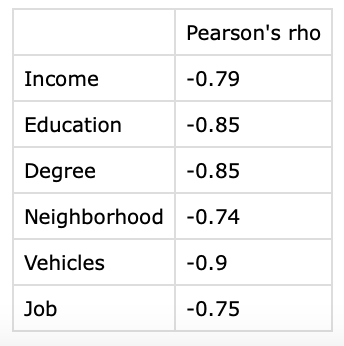


**Supplementary Table 10: Region-by-region effects using standard linear regression after PCA in grey matter volumes.** Without shrinkage penalization and without multi-output modeling, we still find left-right divergences in brain-SES associations using Pearson’s correlation across effects of homologous regions from the left and right brain hemisphere.


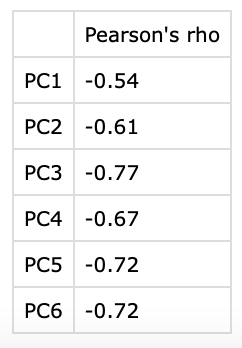


**Supplementary Table 11: Tract-by-tract effects using standard linear regression after PCA in white matter microstructure.** Without shrinkage penalization and without multi-output modeling, we still find left-right divergences in brain-SES associations using Pearson’s correlation across effects of homologous tracts from the left and right brain hemisphere.


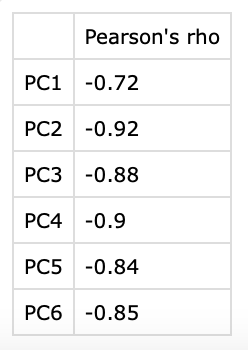


**Supplementary Table 12: Benchmarking analysis of different machine-learning algorithms regarding in-sample prediction from grey matter volumes**

**
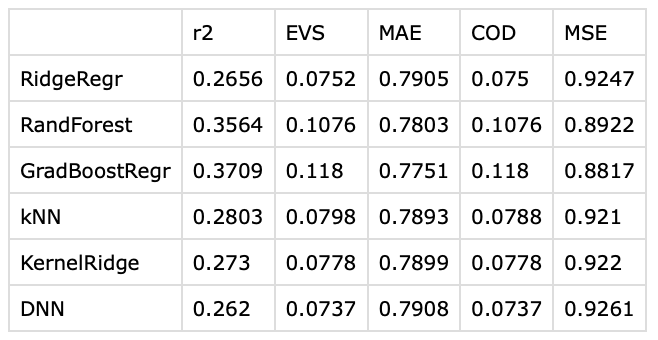
**

RidgeRegr=multivariate multi-output ridge regression; RandForest=random forest algorithms; GradBoostRetr=gradient-boosting trees algorithm; kNN=k-nearest-neighbor algorithms; KernelRidge=kernel-augmented ridge regression; DNN=deep learning algorithms; r2=Pearson’s correlation coefficient, EVS=explained variance score, MAE=mean-absolute error, COD=coefficient of determination, MSE=mean-squared error (see Supplementary Material and Methods for details)

**Supplementary Table 13: Benchmarking analysis of different machine-learning algorithms regarding in-sample prediction from white matter microstructure**

**
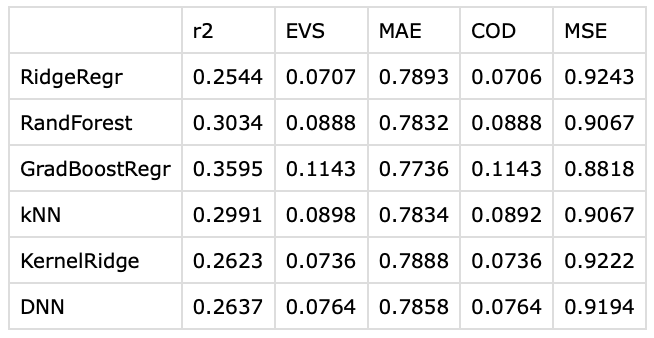
**

RidgeRegr=multivariate multi-output ridge regression; RandForest=random forest algorithms; GradBoostRetr=gradient-boosting trees algorithm; kNN=k-nearest-neighbor algorithms; KernelRidge=kernel-augmented ridge regression; DNN=deep learning algorithms; r2=Pearson’s correlation coefficient, EVS=explained variance score, MAE=mean-absolute error, COD=coefficient of determination, MSE=mean-squared error (see Supplementary Material and Methods for details)

**Supplementary Table 14**

| action | focus | psychosis |
| --- | --- | --- |
| adaptation | gaze | reading |
| addiction | goal | reasoning |
| anticipation | hyperactivity | recall |
| anxiety | imagery | recognition |
| arousal | impulsivity | rehearsal |
| association | induction | reinforcement learning |
| attention | inference | response inhibition |
| autobiographical memory | inhibition | response selection |
| balance | insight | retention |
| belief | integration | retrieval |
| categorization | intelligence | reward anticipation |
| cognitive control | intention | rhythm |
| communication | interference | risk |
| competition | judgment | rule |
| concept | knowledge | salience |
| consciousness | language | search |
| consolidation | language comprehension | selective attention |
| context | learning | semantic memory |
| coordination | listening | sentence comprehension |
| decision | localization | skill |
| decision making | loss | sleep |
| detection | maintenance | social cognition |
| discrimination | manipulation | spatial attention |
| distraction | meaning | speech perception |
| eating | memory | speech production |
| efficiency | memory retrieval | strategy |
| effort | mental imagery | strength |
| emotion | monitoring | stress |
| emotion regulation | mood | sustained attention |
| empathy | morphology | task difficulty |
| encoding | motor control | thought |
| episodic memory | movement | uncertainty |
| expectancy | multisensory | updating |
| expertise | naming | utility |
| extinction | navigation | valence |
| face recognition | object recognition | verbal fluency |
| facial expression | pain | visual attention |
| familiarity | perception | visual perception |
| fear | planning | word recognition |
| fixation | priming | working memory |

List of the 123 terms that were included in the functional association analyses based on the Neurosynth database.
